# The Role of Gut Microbiota and Microbiota-Related Serum Metabolites in the Progression of Diabetic Kidney Disease

**DOI:** 10.3389/fphar.2021.757508

**Published:** 2021-11-24

**Authors:** Qing Zhang, Yanmei Zhang, Lu Zeng, Guowei Chen, La Zhang, Meifang Liu, Hongqin Sheng, Xiaoxuan Hu, Jingxu Su, Duo Zhang, Fuhua Lu, Xusheng Liu, Lei Zhang

**Affiliations:** ^1^ The Second Clinical Medical College, Guangzhou University of Chinese Medicine, Guangzhou, China; ^2^ State Key Laboratory of Dampness Syndrome of Chinese Medicine, Guangdong Provincial Key Laboratory of Clinical Research on Traditional Chinese Medicine Syndrome, The Second Affiliated Hospital of Guangzhou University of Chinese Medicine, Guangzhou, China

**Keywords:** diabetic kidney disease, gut microbiota, serum metabolites, phenylalanine metabolic pathway, tryptophan metabolic pathway, G_Abiotrophia, G_norank_f_Peptococcaceae, G_Lachnospiraceae_NC2004_Group running title

## Abstract

**Objective:** Diabetic kidney disease (DKD) has become the major cause of end-stage renal disease (ESRD) associated with the progression of renal fibrosis. As gut microbiota dysbiosis is closely related to renal damage and fibrosis, we investigated the role of gut microbiota and microbiota-related serum metabolites in DKD progression in this study.

**Methods:** Fecal and serum samples obtained from predialysis DKD patients from January 2017 to December 2019 were detected using 16S rRNA gene sequencing and liquid chromatography-mass spectrometry, respectively. Forty-one predialysis patients were divided into two groups according to their estimated glomerular filtration rate (eGFR): the DKD non-ESRD group (eGFR ≥ 15 ml/min/1.73 m^2^) (n = 22), and the DKD ESRD group (eGFR < 15 ml/min/1.73 m^2^) (n = 19). The metabolic pathways related to differential serum metabolites were obtained by the KEGG pathway analysis. Differences between the two groups relative to gut microbiota profiles and serum metabolites were investigated, and associations between gut microbiota and metabolite concentrations were assessed. Correlations between clinical indicators and both microbiota-related metabolites and gut microbiota were calculated by Spearman rank correlation coefficient and visualized by heatmap.

**Results:** Eleven different intestinal floras and 239 different serum metabolites were identified between the two groups. Of 239 serum metabolites, 192 related to the 11 different intestinal flora were mainly enriched in six metabolic pathways, among which, phenylalanine and tryptophan metabolic pathways were most associated with DKD progression. Four microbiota-related metabolites in the phenylalanine metabolic pathway [hippuric acid (HA), L-(−)-3-phenylactic acid, *trans*-3-hydroxy-cinnamate, and dihydro-3-coumaric acid] and indole-3 acetic acid (IAA) in the tryptophan metabolic pathway positively correlated with DKD progression, whereas L-tryptophan in the tryptophan metabolic pathway had a negative correlation. Intestinal flora *g_Abiotrophia* and *g_norank_f_Peptococcaceae* were positively correlated with the increase in renal function indicators and serum metabolite HA. *G_Lachnospiraceae_NC2004_Group* was negatively correlated with the increase in renal function indicators and serum metabolites [L-(−)-3-phenyllactic acid and IAA].

**Conclusions:** This study highlights the interaction among gut microbiota, serum metabolites, and clinical indicators in predialysis DKD patients, and provides new insights into the role of gut microbiota and microbiota-related serum metabolites that were enriched in the phenylalanine and tryptophan metabolic pathways, which correlated with the progression of DKD.

## Introduction

Diabetic kidney disease (DKD) reflects one of the most common microvascular complications of diabetes, typically characterized by albuminuria or reduced estimated glomerular filtration rate (eGFR) (de Boer et al., 2011; Afkarian et al., 2016). Although advances have occurred in the clinical treatment of DKD, consisting of strict control of blood glucose and blood pressure, and the widely prescribed angiotensin-converting enzyme inhibitors (ACEI) and angiotensin II receptor antagonists (ARB), renal damage can progress (Umanath and Lewis, 2018) with interstitial fibrosis and glomerulosclerosis, and DKD remains the major cause of end-stage renal disease (ESRD) (Ma et al., 2019). Therefore, it is necessary and urgent to elucidate the mechanism of renal fibrosis in DKD and find new biomarkers or targets associated with the progressive renal function decline in DKD patients.

According to the “gut–kidney axis” hypothesis, dysregulation of intestinal microbiota irritates renal tissue through uremic toxins, causing systemic micro-inflammation, renal injury, and fibrosis (Ramezani and Raj, 2014). Recent studies reported that intestinal microbiota has emerged as a pivotal regulator of DKD occurrence in patient with diabetes (Andrade-Oliveira et al., 2015) (Tao et al., 2019). In diabetic patients, the intestinal flora dysbiosis causes intestinal mucosal barrier damage, allowing gut-derived uremic toxins to enter the systemic circulation, which in turn incites an inflammatory response and oxidative stress, and results in insulin resistance, β-cell dysfunction, and kidney injury (Koppe et al., 2018). Age- and gender-matched DKD patients had lower intestinal Prevotella_9 than diabetic patients without kidney disease (Tao et al., 2019), which can produce short-chain fatty acids and reduce the inflammatory reaction of kidney injury. The abundance of Firmicutes in DKD is lower, whereas the abundance of Proteobacteria is higher than that of healthy people and diabetics without renal disease. As inflammation, oxidative stress, and insulin resistance are involved in the renal fibrosis in DKD, then contribute to the development and progression of DKD (Parwani and Mandal, 2020; Rayego-Mateos et al., 2020), therefore, we hypothesized that alteration in intestinal flora may play a crucial role in the progression of DKD to ESRD.

Metabolomics is a powerful tool to screen for changes in metabolic profiles and to characterize mechanisms of pathological changes (Dumas, 2012; Kwan et al., 2020). It can identify and analyze small-molecule metabolites (<1,500 Da) in serum, urine, and feces. In DKD patients, metabolomics plays a great role in screening metabolic biomarkers and detecting abnormal changes in their living organisms (Eid et al., 2019; Kwan et al., 2020). Some urinary metabolites such as indoxyl sulfate, creatinine, and the methoxylated form of phenylacetic acid have been associated with low eGFR in nonproteinuric type 2 diabetes mellitus (Ng et al., 2012). Serum metabolites, such as creatinine, aspartic acid, γ-butyrobetaine, citrulline, symmetric dimethylarginine, kynurenine, azelaic acid, and galactaric acid, can distinguish between DKD with macroalbuminuria and diabetic patients without albuminuria (Hirayama et al., 2012).

The large and complex microbial community in the human intestinal tract has a profound impact on human metabolic phenotype. As the mediator of the interaction between intestinal flora and diseases, the metabolites can more directly show the relationship between intestinal flora and diseases. Ma et al. combined 16S rRNA and metabolomics technology and determined that flora-metabolites combined with the flora-bacteria might represent a new detection method for breast cancer (Ma et al., 2020). Evidence has confirmed that it is possible to characterize the relationship between intestinal microecology and disease by associating intestinal microflora with metabolites via multiomics-integrated methods. Gut microbiota and related metabolites, such as tryptophan metabolism and polyamine metabolism, have been reported to mediate renal fibrosis in the rat model of CKD (Feng et al., 2019; Hu et al., 2020b; Liu JR. et al., 2021). However, very few studies have explored the role of gut microbiota and microbiota-related metabolites in the DKD progression.

In the present study, we aimed to investigate gut microbiota profiles and serum metabolic characteristics in predialysis DKD patients that were associated with DKD progression and to explore the correlation between intestinal flora and metabolic disorders using multiomics technology of 16S rRNA gene sequencing and metabolomics.

## Materials and Methods

### Study Design

This study detected fecal and serum samples of 41 predialysis DKD patients from January 2017 to December 2019 in Guangdong Provincial Hospital of Chinese Medicine. The patients were divided into two groups according to their renal function (eGFR): the DKD non-ESRD group (GFR ≥ 15 ml/min/1.73 m^2^)_,_ and the DKD ESRD group (eGFR < 15 ml/min/1.73 m^2^). The study protocol was approved by the Institutional Ethics Committee of Guangdong Provincial Hospital of Chinese Medicine (No. ZE2020-193-01), and informed consent was obtained before sample collection.

### Patients

Serum and fecal samples of predialysis DKD patients were obtained from the biological resource bank of Guangdong Province Hospital of Chinese Medicine. Estimated glomerular filtration rate (eGFR) was calculated using the chronic kidney disease epidemiology collaboration (CKD-EPI) equation (National Kidney Foundation, 2002).

#### Inclusion and Exclusion Criteria

The inclusion criteria were age from 18 to 85 years, diagnosis of DKD, and nonrenal replacement therapy. Note: renal replacement therapy refers to hemodialysis, peritoneal dialysis, and renal transplantation.

The exclusion criteria were incomplete clinical data, concomitant active malignant tumor, pulmonary infection, acute coronary heart disease, and other acute complications, antibiotics or probiotics having been taken 3 months prior to sample collection, and corticosteroid or immunosuppressive therapy prior to sample collection.

### Sample Collection

At least 1 g of fresh feces was collected by sterilized cotton swabs in a special fecal collection tube. Blood samples were collected by venipuncture in EDTA tubes; serum was separated by centrifugation. Feces and serum were stored immediately at −80 °C until further processing.

### 16s rRNA Sequencing and Data Processing

#### DNA Extraction and PCR Amplification

Microbial community genomic DNA was extracted from feces samples using the E. Z.N.A.^®^ soil DNA Kit (Omega Bio-tek, Norcross, GA, United States). The DNA extract was checked on 1% agarose gel, and DNA concentration and purity were determined with NanoDrop 2000 UV-vis spectrophotometer (Thermo Scientific, Wilmington, DE, United States). The hypervariable region V3–V4 of the bacterial 16S rRNA gene were amplified with primer pairs 338F (5′-ACT​CCT​ACG​GGA​GGC​AGC​AG-3′) and 806R (5′-GGACTACHVGGGTWTCTAAT-3′) by an ABI GeneAmp^®^ 9700 PCR thermocycler (ABI, CA, United States). The PCR product was extracted from 2% agarose gel and purified using the AxyPrep DNA Gel Extraction Kit (Axygen Biosciences, Union City, CA, United States) and quantified using Quantus™ Fluorometer (Promega, United States).

#### Illumina MiSeq Sequencing

Purified amplicons were pooled in equimolar and paired-end sequenced on an Illumina MiSeq PE300 platform/NovaSeq PE250 platform (Illumina, San Diego, CA, United States) according to the standard protocols by Majorbio Bio-Pharm Technology Co. Ltd. (Shanghai, China).

#### Processing of Sequencing Data

The raw 16S rRNA gene sequencing reads were demultiplexed, quality-filtered by fast version 0.20.0 (Chen et al., 2018) and merged by FLASH version 1.2.7 (Magoc and Salzberg, 2011). Operational taxonomic units (OTUs) with 97% similarity cutoff were clustered using UPARSE version 7.1 (Edgar, 2013), and chimeric sequences were identified and removed. The taxonomy of each OTU representative sequence was analyzed by RDP Classifier version 2.2 (Wang et al., 2007) against the 16S rRNA database (e.g., Silva v132) using confidence threshold of 0.7.

### Liquid Chromatography-Mass Spectrometry Detection

#### Metabolite Extraction

The metabolites were extracted from 100 µl of liquid sample and treated by high-throughput tissue crusher Wonbio-96c (Shanghai Wanbo Biotechnology Co., Ltd.), then followed by ultrasound for 30 min. After centrifugation, the supernatant was carefully transferred to sample vials for LC-MS/MS analysis.

#### UPLC-MS/MS Analysis

Chromatographic separation of the metabolites was performed on an ExionLC™ AD system (AB Sciex, United States) equipped with an ACQUITY UPLC HSS T3 column (Waters, Milford, CT, United States). The UPLC system was coupled to a quadrupole-time-of-flight mass spectrometer (Triple TOF™5600+, AB Sciex, United States) equipped with an electrospray ionization (ESI) source operating in positive mode and negative mode. Data acquisition was performed with the data-dependent acquisition (DDA) mode.

#### Data Preprocessing and Annotation

The raw data were imported into the Progenesis QI 2.3 (Nonlinear Dynamics, Waters, United States) for peak detection and alignment. Mass spectra of these metabolic features were identified by using the accurate mass, MS/MS fragments spectra, and isotope ratio difference with search in reliable biochemical databases, such as the Human Metabolome Database (http://www.hmdb.ca/) and Metlin database (https://metlin.scripps.edu/).

### Statistical Analysis

Results were expressed as frequencies and percentages for categorical variables, mean ± SD for continuous normally distributed variables, and median (interquartile range, IQRs) for continuous variables that were not normally distributed. Categorical variables for the patient characteristics were compared using the chi-square test or Fisher’s exact test, and the continuous variables were tested with *t*-test or nonparametric Wilcoxon rank sum test. All analyses were performed using the SPSS version 19.0 (SPSS Inc., Chicago, IL, United States) and “ropls” (Version 1.6.2, http://bioconductor.org/packages/release/bioc/html/ropls.html) R package from Bioconductor on the Majorbio Cloud Platform (www.majorbio.com) with a two-sided *p*-value less than 0.05 considered significant.

#### Gut Microbiota Analysis

We used rarefaction curves and species accumulation curves to ensure that the sample size or sequencing depth reached saturation in our study. Gut microbiota alpha diversity index (Shannon index, Chao index) was analyzed on mothur software (version 1.30.1, http://www.mothur.org/), tested by nonparametric Wilcoxon rank sum test, and *p* < 0.05 was considered statistically significant. Beta diversity measured the difference in OTU composition between different samples and was assessed using partial least squares discriminant analysis (PLS-DA), which is a supervised analysis suitable for high-dimensional data. The corresponding statistical significance of the beta diversity was measured separately by ANOSIM.

Compositional differences between the two groups from the phylum to genus level were tested with nonparametric Wilcoxon rank-sum test. Variation at the taxonomic level was determined by linear discriminant analysis (LDA) effect size (LDA score >1, *p* < 0.05) calculated by the LEfSe software (http://huttenhower.sph.harvard.edu/). The correlation between biochemical indicators and various microbes was calculated by Spearman rank correlation coefficient and visualized by heatmap in R using the “heatmap” package.

#### Metabolomic Analysis

Orthogonal partial least squares discriminate analysis (OPLS-DA) was used for statistical analysis to determine global metabolic changes between comparable groups. All metabolite variables were scaled to Pareto scaling prior to conducting the OPLS-DA. The model validity was evaluated from model parameters *R* ^2^ and Q^2^, which provided information for the interpretability and predictability, respectively, of the model and avoided the risk of overfitting. Variable importance in the projection (VIP) was calculated in the OPLS-DA model. Values of *p* were estimated with paired Student’s t-test on single-dimensional statistical analysis. Metabolites with VIP >1 and *p* < 0.05 were considered statistically significant. We used the area under the receiver operating characteristic (ROC) curve to assess the accuracy of the metabolites in predicting DKD progression.

Differential metabolites between the two groups were summarized and mapped into their biochemical pathways through metabolic enrichment and pathway analysis based on database search (KEGG, http://www.genome.jp/kegg/). These metabolites could be classified according to the pathways they involved or the functions they performed. Enrichment analysis was used to analyze a group of metabolites in a function node whether it appears or not. Scipy. stats (Python packages) (https://docs.scipy.org/doc/scipy/) was exploited to identify statistically significantly enriched pathways using Fisher’s exact test.

The correlation between differential metabolites and various microbes was calculated by Spearman rank correlation coefficient and visualized by heatmap in R software using the “heatmap” package.

## Results

### Clinical and Biochemical Characteristics

Samples from 41 predialysis patients were divided into the DKD non-ESRD group (eGFR ≥ 15 ml/min/1.73 m^2^ group) (n = 22) or the DKD ESRD group (eGFR < 15 ml/min/1.73 m^2^ group) (n = 19), with mean ages of 69.63 ± 13.01 and 61.89 ± 9.85 in the two groups, respectively. Compared with the DKD non-ESRD group, the levels of serum creatinine and blood urea nitrogen were higher in the DKD ESRD group (*p* < 0.001). There were no significant differences in other baseline indicators between the two groups (Table 1).

**TABLE 1:** Patient clinical and biochemical characteristics.

Characteristics	Total (n = 41)	DKD non-ESRD group (n = 22)	DKD ESRD group (n = 19)
Male, n. (%)	27 (65.85%)	16 (72.73%)	11 (57.89%)
Age (years)	65.39 ± 11.38	69.63 ± 13.01	61.89 ± 9.85
Blood pressures (mmHg)			
Systolic	158.49 ± 22.36	160.90 ± 21.98	156.00 ± 23.48
Diastolic	82.63 ± 11.91	84.22 ± 13.1	79.47 ± 10.16
SCr (µmol/L)	372.90 ± 232.59	189.70 ± 74.64*	577.02 ± 164.35
eGFR (ml/min/1.73 m^2^)	22.91 ± 18.81	36.01 ± 16.77*	7.78 ± 2.37
24hU-pro (g/24 h) (IQR)	3.82 (1.38, 5.16)	3.00 (0.95, 5.7)	3.83 (1.94, 5.01)
HbA1c (%)	6.40 (5.56, 7.45)	6.90 (5.70, 7.60)	5.85 (5.38, 7.10)
UA (µmol/L) (IQR)	476.50 (370.00, 574.00)	451.00 (357.50, 574.00)	508.00 (434.00, 587.00)
BUN (mmol/L) (IQR)	17.70 (11.01, 24.47)	11.23 (8.36, 13.52)*	23.47 (20.48, 29.98)
Triglycerides (mmol/L) (IQR)	1.75 (1.16, 2.37)	1.63 (0.88, 2.03)	2.11 (1.42, 2.62)
Cholesterol (mmol/L)	5.18 ± 1.77	5.34 ± 1.82	4.97 ± 1.86
HDL (mmol/L)	1.13 ± 0.35	1.33 ± 0.59	1.03 ± 0.35
LDL (mmol/L)	3.42 ± 1.60	3.66 ± 1.71	3.13 ± 1.50
Serum albumin (g/L)	35.23 ± 5.65	34.44 ± 6.72	36.10 ± 3.84
AST (U/L) (IQR)	16.00 (13.00, 20.00)	17.00 (13.00, 21.00)	16.00 (11.00, 20.50)
ALT (U/L) (IQR)	12.00 (9.00, 21.00)	14.00 (10.75, 21.25)	9.00 (7.00, 16.00)

Note. Abbreviations: SCr, serum creatinine; eGFR, estimated glomerular filtration rate; 24hU-pro, 24-h urinary protein quantity; UA, uric acid; BUN, blood urea nitrogen; LDL, low-density lipoprotein; HDL, high-density lipoprotein; AST, glutamic oxaloacetic transaminase; ALT, alanine aminotransferase; IQR, interquartile range; DKD, diabetic kidney disease; ESRD, end-stage renal disease.

*p < 0.05 vs. DKD ESRD group.

### Gut Microbiota Analysis

#### Alpha Diversity and Beta Diversity

**FIGURE 1:**
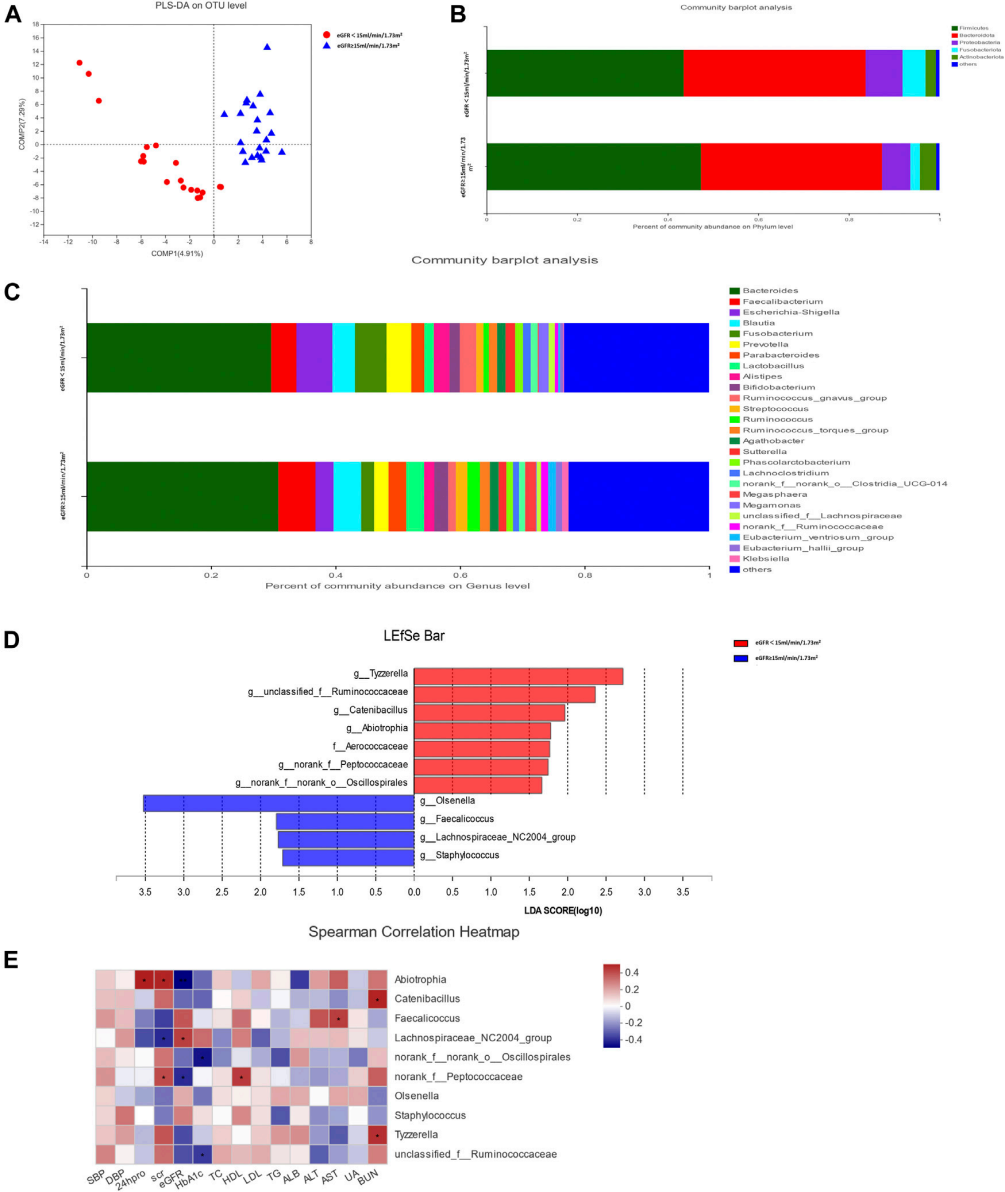
Gut microbiota analysis between groups in diabetic kidney disease (DKD) patients. **(A)** Analysis of beta diversity using partial least squares discriminant analysis (PLS-DA) revealed that the microbial composition between groups was significantly different. One dot in the figure represents one sample. **(B)** The composition and relative abundance of intestinal microbiota at the phylum level. **(C)** The composition and relative abundance of intestinal microbiota at the genus level. **(D)** Linear discriminant analysis (LDA) effect size (LEfSe) bar plot. The LEfSe was used to identify the species that significantly differed between groups. **(E)** Correlation heatmap analysis between the intestinal flora and clinical indicators. Red represents a positive correlation, and blue represents a negative correlation.

The rarefaction curve indicated that the sequencing depth of each sample approached the expected level (Supplementary Figure S1). Alpha diversity analysis revealed no significant difference in gut microbiota diversity between each group based on Chao and Shannon indices (*p* > 0.05) (Supplementary Figure S2). The result of beta diversity based on PLS-DA showed that the microbial composition between groups was significantly different (Figure 1A).

#### Relative Abundance of Species

The relative abundance percentage of gut microbiota at the phylum and genus level was analyzed to identify taxa that could display significant differences in the two groups. At the phylum level, Firmicutes and Bacteroidota were the most abundant, and their mean relative abundance were similar in the DKD ESRD and DKD non-ESRD groups, accounting for 44.02 ± 14.30% and 39.05 ± 16.09% in the DKD ESRD group, and 47.78 ± 19.61% and 39.58 ± 18.90% in the DKD non-ESRD group, respectively (Figure 1B). At the genus level, *Bacteroides* represented the highest abundance of OTU in the two groups. The mean relative abundance for *Bacteroides* was similar in the two groups, accounting for 28.74 ± 17.60% in the DKD ESRD group and 30.33 ± 22.34% in the DKD non-ESRD group. The mean relative abundance for *Faecalibacterium* was also similar in the two groups, accounting for 3.99 ± 2.91% in the DKD ESRD group and 5.84 ± 7.04% in the DKD non-ESRD group. Likewise, other gut microbiota, such as *Blautia*, *Escherichia–Shigella*, *Fusobacterium*, etc., did not demonstrate a significant difference in their relative abundance in either DKD ESRD or DKD non-ESRD group (Figure 1C).

#### Different Species Analysis

Based on the LDA selection, 10 differential intestinal flora at the genus level and one differential intestinal flora at the family level were identified in the fecal samples between the two groups (LDA > 1, *p* < 0.05). Compared with the DKD non-ESRD group, the levels of *g_Tyzzerella*, *g_Ruminococcaceae*, *g_Catenibacillus*, *g_Abiotrophia*, *g_norank_f_Peptococcaceae*, *g_norank_f_norank_o_Oscillospirales*, and f_Aerococcaceae were significantly higher, and the levels of *g_Olsenella*, *g_Faecalicoccus*, *g_Lachnospiraceae_NC2004_group*, and *g_Staphylococcus* were significantly lower in the DKD ESRD group (Figure 1D).

#### Correlation Analysis Between the Intestinal Flora and Clinical Indicators

Correlation analysis of the 10 differential intestinal floras at the genus level and the clinical indicators of the patient showed that *g_Abiotrophia* had a positive correlation with serum creatinine and 24-h urinary protein, and negative correlation with eGFR (*p* < 0.05). *G_norank_f_Peptococcaceae* had a positive correlation with serum creatinine and a negative correlation with eGFR (*p* < 0.05). In contrast, g*_Lachnospiraceae_NC2004_group* had a strong negative correlation with serum creatinine and a positive correlation with eGFR (*p* < 0.05). *G_norank_f_norank_o_Oscillospirales* and *g_unclassified_f_Ruminococcaceae* had a strong negative correlation with glycosylated hemoglobin (HbA1c) (*p* < 0.05) (Figure 1E).

### Serum Metabolomics Analysis

#### Different Serum Metabolites Between Groups

**FIGURE 2:**
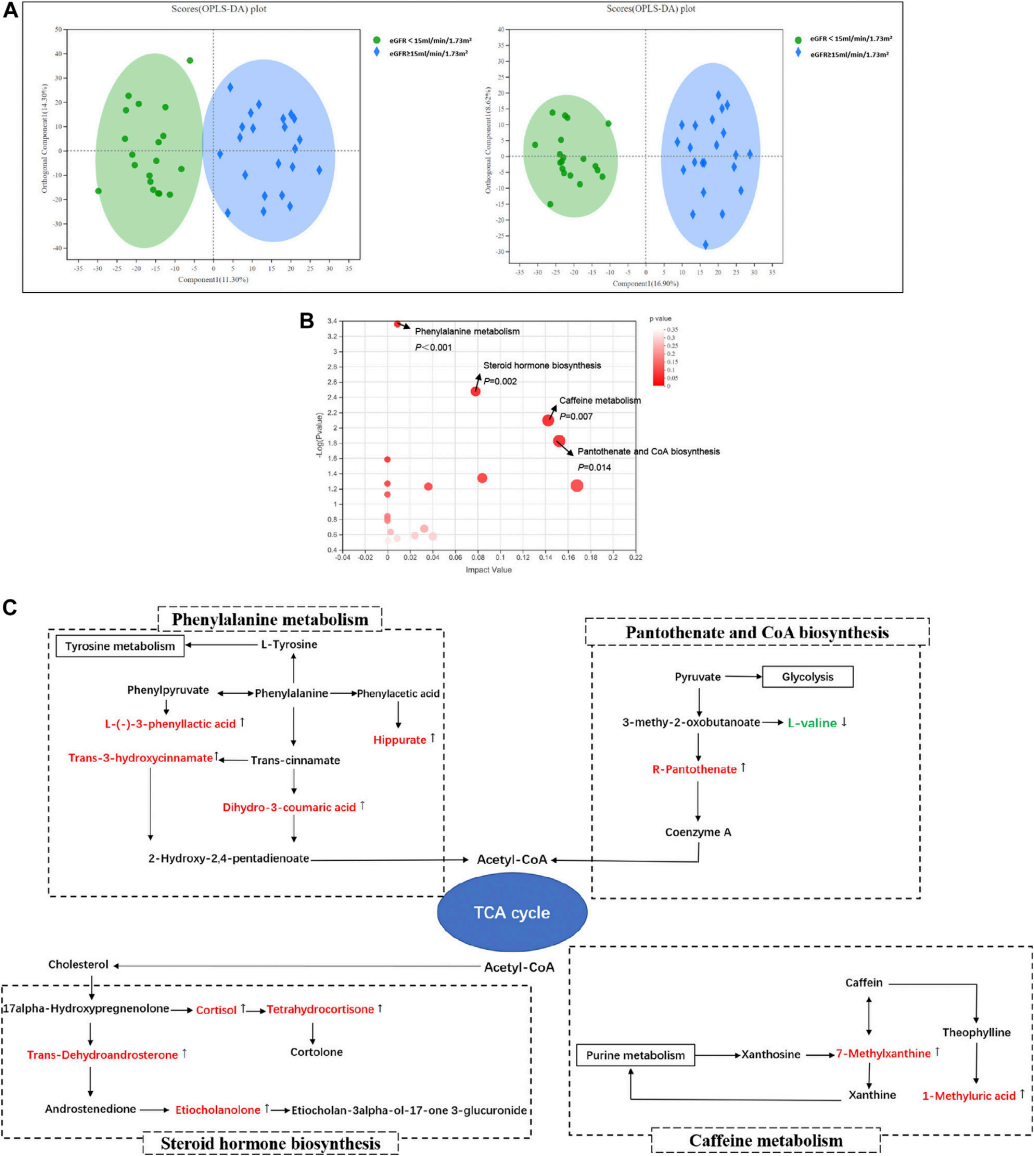
Serum metabolomics analysis between groups in DKD patients. **(A)** Orthogonal partial least squares discriminate analysis (OPLS-DA) score plots of serum metabolic profiling in positive mode (left) and negative mode (right); positive mode: R^2^X = 0.256, R^2^Y = 0.857, Q^2^ = 0.702; negative mode: R^2^X = 0.255, R^2^Y = 0.930, Q^2^ = 0.694; **(B)** The bubble plot of KEGG analysis. Each bubble in the figure represents a KEGG pathway. The horizontal axis indicates the relative importance of metabolites in the pathway, and the vertical axis indicates the statistical significance of metabolites in the pathway; **(C)** Schematic diagram of phenylalanine metabolism, caffein metabolism, pantothenic acid and coenzyme A biosynthesis, steroid hormone biosynthesis, and their relevant differential metabolite alterations during DKD progression. The upregulated metabolites in the DKD ESRD group were labeled with red and downregulated metabolites in the DKD ESRD group with green.

The profile of metabolites showed definite separation between the two groups in OPLS-DA score plots (Figure 2A). Different serum metabolites (239) were obtained (VIP > 1, *p* < 0.05) based on the OPLS-DA model (Supplementary Table S1) and were included in further analysis. Nineteen metabolites, with VIP >3 and *p* < 0.05, had a higher concentration in the DKD ESRD group and are shown in Table 2.

**TABLE 2:** Differential serum metabolites between groups in DKD patients (VIP > 3 and *p* < 0.05).

Metabolite	Compound ID	M/Z	Metabolite changes	VIP_	FC	AUC	95% CI
5a-Androst-3-en-17-one	HMDB0006046	273.220	↑	4.305	4.846	0.931	[0.86, 1]
*Trans*-Dehydroandrosterone	C01227	289.215	↑	4.284	2.238	0.938	[0.869, 1]
Tryptophyl-cysteine	HMDB0029080	330.085	↑	4.212	2.471	0.997	[0.991,1]
5-Androstene-3b,16b,17a-triol	HMDB0000523	307.226	↑	3.992	3.513	0.931	[0.859, 1]
Oxindole	C12312	134.059	↑	3.931	2.467	0.913	[0.828, 0.999]
3,4,5-Trihydroxy-6-[(3-methy lbut-enoyl)oxy]oxane-2-carboxylic acid	HMDB0128920	318.117	↑	3.823	3.118	0.877	[0.766, 0.988]
6-Dehydrotestosterone	—	287.200	↑	3.779	2.545	0.845	[0.719, 0.971]
(2E,4E)-2,7-Dimethyl-2,4-octadienedioic acid	HMDB0034099	181.085	↑	3.528	1.761	0.931	[0.852, 1]
O-Adipoylcarnitine	HMDB0061677	290.159	↑	3.523	1.380	0.965	[0.921, 1]
Mono-(2-ethyl-5-carboxypentyl) phthalate	HMDB0094647	331.114	↑	3.508	2.567	0.881	[0.781, 0.982]
Atrolactic acid	C05584	167.069	↑	3.457	1.616	0.925	[0.848, 1]
Benzenebutanoic acid	HMDB0000543	165.091	↑	3.411	2.310	0.915	[0.830, 1]
3,5-Cyclo-5alpha,17alpha-pregn-20-yne-6beta,17-diol	C15468	315.231	↑	3.396	2.162	0.929	[0.850, 1]
Indoleacetyl glutamine	HMDB0013240	304.128	↑	3.202	1.618	0.813	[0.669, 0.958]
{[3-(2,5-Dihydroxyphenyl) prop-2-en-1-yl]oxy}sulfonic acid	HMDB0134083	291.018	↑	3.191	4.360	0.975	[0.940, 1]
N-Acetylproline	HMDB0094701	199.107	↑	3.183	1.514	0.922	[0.828, 1]
1-Methyluric acid	C16359/HMDB0003099	183.050	↑	3.182	2.714	0.85	[0.732, 0.968]
3,4,5-Trimethoxyphenyl acetate	HMDB0031722	209.080	↑	3.056	1.473	0.872	[0.756, 0.988]
3-Indole carboxylic acid glucuronide	HMDB0013189	336.071	↑	3.011	2.243	0.959	[0.902, 1]

Note. Abbreviations: M/Z, mass-to-charge ratio; VIP, the variable importance in projection; FC, fold change; AUC, area under curve. Metabolite changes in the DKD ESRD group are shown as (↑) for increase or (↓) for decrease. Compound ID starting with C is from the KEGG database. Compound ID starting with HMDB is from the Human Metabolome Database.

#### Pathway Enrichment of Different Metabolites Analysis

Enrichment pathway analysis of 239 different metabolites showed that phenylalanine metabolism, caffeine metabolism, pantothenate and CoA biosynthesis, and steroid hormone biosynthesis were involved in the DKD progression (Figure 2B). Correlation networks were drawn to show the four metabolic pathways as well as the changes in their relevant different metabolites between groups (Figure 2C).

Compared with the DKD non-ESRD group, the concentration of hippuric acid (HA), L-(−)-3-phenyllactic acid, dihydro-3-coumaric acid, and *trans*-3-hydroxycinnamate enriched in the phenylalanine metabolism pathway, 7-methylxanthine and 1-methyluric acid enriched in the caffeine metabolism pathway, R-pantothenate enriched in the pantothenate and CoA biosynthesis pathway, and *trans*-dehydroandrosterone, cortisol, tetrahydrocortisone, and etiocholanolone enriched in the steroid hormone biosynthesis pathway was higher in the DKD ESRD group. In contrast, L-valine enriched in the pantothenate and CoA biosynthesis pathway had a lower concentration in the DKD ESRD group.

### Integrating Multiomics Analysis

#### Microbiota-Related Metabolites Analysis

Interomics correlation analyses were used to further explore the correlation between the gut microbiota and metabolome composition. Based on the intestinal flora participating in the metabolism of the host, the correlation of 11 between-group different intestinal floras and 239 between-group different metabolites was calculated and illustrated in the form of a correlation coefficient matrix heat map (Supplementary Figure S3). Microbiota-related metabolites (192) were screened out based on *p* < 0.05 according to previous studies (Walker et al., 2016; Nunez Lopez et al., 2019; Wei et al., 2020; Wu et al., 2021) (Supplementary Table S2).

#### Pathway Enrichment Analysis of Microbiota-Related Metabolites

**FIGURE 3:**
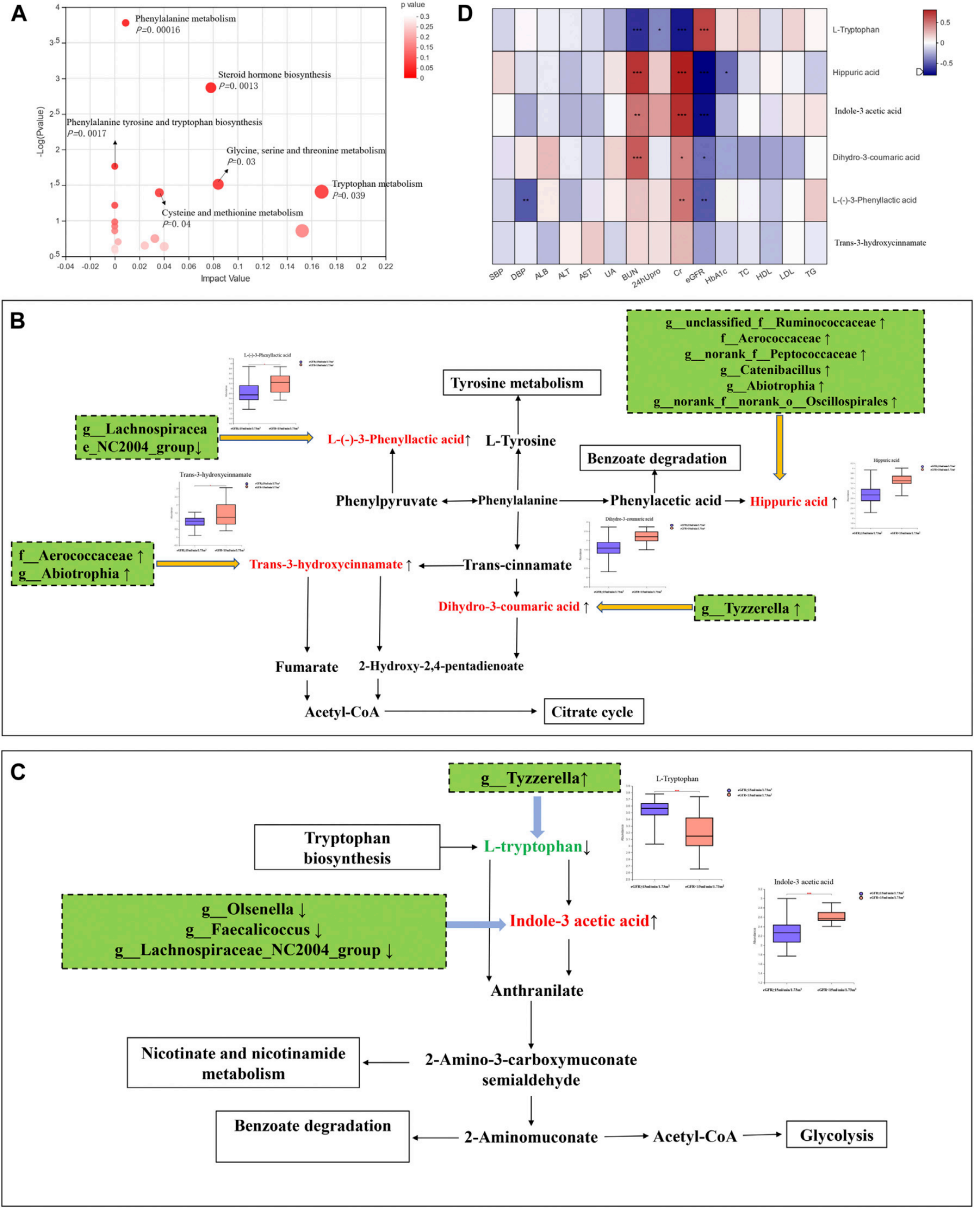
Integrating multiomics analysis. **(A)** The bubble plot of KEGG analysis. Each bubble in the figure represents a KEGG pathway. The horizontal axis indicates the relative importance of metabolites in the pathway, and the vertical axis indicates the statistical significance of metabolites in the pathway. **(B)** Metabolic pathway map of phenylalanine metabolism. The metabolites shown in red are the differential metabolites that are highly expressed in the DKD ESRD group. Other related metabolic pathways were expressed in solid wire frame. **(C)** Metabolic pathway map of tryptophan metabolism. The metabolites expressed in red and green were statistically different between the two groups. Red metabolites express upregulated in the DKD ESRD group; green metabolites express downregulated in the DKD ESRD group. Other related metabolic pathways were expressed in solid wire frame. **(D)** Correlation analysis between different metabolites in enrichment pathway and clinical indicators.

There were 192 microbiota-related metabolites submitted to the KEGG website for metabolic pathway enrichment analysis. Six enriched pathways with significant differences between groups were identified (Figure 3A), among which, the phenylalanine and tryptophan metabolic pathways were selected as the pathways most associated with DKD progression, according to the impact value and *p*-value in the KEGG analysis. Correlation networks between the intestinal flora and microbiota-related metabolites enriched on the two pathways were constructed (Figures 3B,C).

Four microbiota-related metabolites enriched on the phenylalanine metabolic pathway [HA, L-(−)-3-phenylactic acid, *trans*-3-hydroxy-cinnamate and dihydro-3-coumaric acid] had higher concentrations in the DKD ESRD group, compared with the other group. Among 11 differential intestinal floras, *g_unclassified_f_Ruminococcaceae*, f_Aerococcaceae, *g_norank_f_Peptococcaceae*, *g_Catenibacillus*, *g_Abiotrophia*, and *g_norank_f_norank_o_Oscillospirales* were positively correlated with HA. *G_Lachnospiraceae_NC2004_Group* was negatively correlated with L-(−)-3-phenylactic acid. *F_Aerococcaceae and g_ Abiotrophia* were positively correlated with *trans*-3-hydroxy-cinnamate. *G_ Tyzzerella* was positively correlated with dihydro-3-coumaric acid (Figure 3B).

As microbiota-related metabolites enriched on the tryptophan metabolic pathway, indole-3 acetic acid (IAA) was highly expressed, and L-tryptophan had low expression in the DKD ESRD group compared with the DKD non-ESRD group. Among 11 differential intestinal floras, *g_Olsenella*, *g_Faecalicoccus*, and *g_Lachnospiraceae_NC2004_Group* were negatively correlated with indole-3 acetic acid. *G_Tyzzerella* was negatively correlated with L-tryptophan (Figure 3C).

#### Correlation Analysis Between Microbiota-Related Metabolites and Clinical Biomarkers

To further verify the role of microbiota-related metabolites enriched on the phenylalanine and tryptophan metabolic pathways in DKD progression, a correlation analysis between the above six microbiota-related metabolites and clinical indicators was undertaken. Consistent with the results of comparison between groups, HA, L-(−)-3-phenyllactic acid, and dihydro-3-coumaric acid in the phenylalanine metabolic pathway and IAA in the tryptophan metabolic pathway were positively correlated with serum creatinine and negatively correlated with eGFR, whereas L-tryptophan in the tryptophan metabolic pathway was opposite (Figure 3D).

## Discussion

In this study, 11 significantly different intestinal flora and 239 significantly different metabolites were identified between the DKD non-ESRD group and the DKD ESRD group. The phenylalanine and tryptophan metabolic pathways were most associated with DKD progression. Four microbiota-related metabolites in the phenylalanine metabolic pathway [HA, L-(−)- 3-phenylactic acid, *trans*-3-hydroxy-cinnamate, dihydro-3-coumaric acid], and IAA in the tryptophan metabolic pathway positively correlated with DKD progression, whereas L-tryptophan in the tryptophan metabolic pathway had a negative correlation. Intestinal flora *g_Abiotrophia* and *g_norank_f_Peptococcaceae*, both of which positively correlated with DKD progression, had a positive correlation with a high level of HA. *G_Lachnospiraceae_NC2004_Group*, which negatively correlated with DKD progression, also had a negative correlation with a high level of IAA and L-(−)-3-phenyllactic acid, simultaneously. In addition, g*_Tyzzerella* was positively correlated with dihydro-3-coumaric acid and negatively correlated with L-tryptophan. *G_unclassified_f_Ruminococcaceae* was positively correlated with HA, but negatively with HbA1c. These results indicated the potential role of specific gut microbiota in the DKD progression associated with the phenylalanine and tryptophan metabolism.

This study identified the phenylalanine metabolic pathway as the one most associated with DKD progression. Three microbiota-related serum metabolites [HA, L-(−)-3-phenylactic acid, and dihydro-3-coumaric acid] in the phenylalanine metabolic pathway were positively correlated with deterioration of renal function in DKD patients. Abnormal phenylalanine metabolism has previously been demonstrated in patients with diabetes (Liu Y. et al., 2019) and type 2 diabetic animal models (Pan et al., 2020). However, its role in the DKD progression remains unclear.

As intermediates of phenylalanine metabolism, HA, which is a common protein-bound uremic toxin (PBUT) in patients with ESRD, is related to the progress of renal fibrosis due to its oxidative stress-associated toxicity (Sun et al., 2020). It is generated from the metabolism of many dietary components including phenylalanine and polyphenolic compounds, such as catechins and cinnamic acid from vegetables, fruit, tea, and coffee (Lees et al., 2013). These compounds are converted into benzoic acid, then further converted into HA, which is excreted in the urine. There has been no study that has investigated the impact of the serum metabolites L-(−)-3-phenylactic acid and dihydro-3-coumaric acid on DKD progression. Previous studies have indicated that they might be involved in the synthesis of HA. L-(−)-3-phenyllactic acid is an organic compound belonging to the class of phenylpropanoic acids, which could be derived from catechins by the colonic microbiota (Olthof et al., 2001). Dihydro-3-coumaric acid, also named 3-hydroxyphenylpropionic acid, belongs to hydroxycinnamic acid derivatives of cinnamic acid. These two metabolites may undergo further metabolism to benzoic acid and finally metabolized to HA (Phipps et al., 1998).

The gut microbiota makes up the largest microecosystem in the human body and is closely related to metabolic disorders in kidney disease. Several studies have reported the relationship between gut microbiota and phenylalanine metabolism in CKD patients (Hu et al., 2020a; Ren et al., 2020; Wu et al., 2020), but the evidence is mainly based on the functional analysis of gut microbiome. Very few studies explored the relationship between gut microbiota and phenylalanine metabolism in DKD patients (Fang et al., 2021). The present research mainly focused on the potential role of gut microbial and protein-bound uremic toxins, such as HA, which originate from the gut microbial metabolism of phenylalanine (Koppe et al., 2018; Fernandes et al., 2019).

Studies have demonstrated the significance of the gut microbiota in contributing to the synthesis of HA in phenylalanine metabolism (Phipps et al., 1998; Olthof et al., 2001; Olthof et al., 2003). For example, perturbation of hippurate levels has often been attributed to gut microbial activity. The phenolic dietary components are metabolized to phenylpropionic acids by the colonic microbiota, and are then absorbed and metabolized in the liver via β-oxidation to produce benzoic acid, before glycine conjugation and excreted as hippurate. It was proposed that type II diabetes is often related to obesity. The change in hippurate levels is due, at least in part, to potential differences in the microbiota as a result of the “obese microbiome,” relative proportion alteration of Firmicutes and Bacteroidetes (Lees et al., 2013).

This study indicated the potential role of intestinal bacteria *g_Abiotrophia* and *g_norank_f_Peptococcaceae* in DKD progression, and their positive correlation with serum HA concentration in DKD, which has not been previously reported. However, an increasing amount of evidence has suggested their involvement in abnormal glucose and lipid metabolism and insulin resistance (Liu D. et al., 2019; Liu YK. et al., 2021; Yuan et al., 2021), supporting our findings and the hypothesis of their role in DKD progression. Furthermore, *g_Abiotrophia* was positively correlated with dihydro-3-coumaric acid, indicating its important role in the synthesis of HA and phenylalanine metabolism.

Consistent with prior studies, *f_Ruminococcaceae* was positively correlated with serum HA concentration in this study. Ruminococcaceae has been considered as a principal short-chain fatty acid-producing bacteria, significantly increased in fecal samples of patients with insulin resistance, and T2D patients compared with healthy subjects (Zhao et al., 2019). Suppressing the growth of Ruminococcaceae has exerted hypoglycemic effects in diabetic animal models (Hu et al., 2019; Zhang et al., 2020). However, no significant correlation between Ruminococcaceae and serum renal function indicators of DKD patients was observed in our study, consistent with prior reports (Lecamwasam et al., 2020). Interestingly, although Ruminococcaceae represented the highest abundance in the fecal sample of DKD patients, the relative abundance of this gut microbe does not change across the stages (1–5) of diabetic CKD.

In concert with previous studies (Debnath et al., 2017; Hasegawa and Inagi, 2021), our study reported the association of the tryptophan metabolic pathway with DKD progression, in which IAA was positively correlated with renal function deterioration, whereas L-tryptophan was just the opposite. Known as a gut-derived protein-bound uremic toxin, IAA is produced by dietary tryptophan metabolism, which stimulates glomerular sclerosis and interstitial fibrosis in the kidneys (Rysz et al., 2021) due to its prooxidant and proinflammatory effect (Gondouin et al., 2013; Dou et al., 2015; Lin et al., 2019). Tryptophan is digested by intestinal bacteria (*E. coli*, *Proteus vulgaris*, *Paracolobactrum coliforme*, *Achromobacter liquefaciens*, and *Bacteroides* spp) to indole (Keszthelyi et al., 2009; Fang et al., 2021), which could evolve into IAA by adding carboxymethyl to the indole ring. Studies have seldom reported the positive effects of pre-, pro-, and synbiotics on the change in serum IAA in CKD patients (Rysz et al., 2021). Therefore, it is meaningful to explore the role of gut microbiota in IAA synthesis to find a new therapeutic strategy. In this study, we observed an inverse correlation between intestinal flora (*g_Lachnospiraceae_NC2004_Group*, *g_Olsenella*, *and g_Faecalicoccus*) and serum IAA, among which, *g_Lachnospiraceae_NC2004_Group* was negatively correlated with L-(−)-3-phenyllactic acid and serum creatinine level, indicating its potential role in the DKD progression via both the phenylalanine and tryptophan metabolic pathways. *G_Lachnospiraceae_NC2004_Group* is a Firmicutes member belonging to *f_Lachnospiraceae*, which was mainly involved in the generation of IAA (Gryp et al., 2020). It is a predominant anaerobic bacteria in the microbial community of healthy populations, producing short-chain fatty acids (Egerton et al., 2020), converting primary bile acids to secondary bile acids and resisting colonization by pathogens (Sorbara et al., 2020). There has been no study that reported an association between *g_Olsenella*, *g_Faecalicoccus*, and IAA synthesis, and their role in DKD progression.

*G_Tyzzerella* was negatively correlated with L-tryptophan and positively correlated with dihydro-3-coumaric acid, indicating its association with the phenylalanine and tryptophan metabolic disorders. As previously reported, *g_Tyzzerella* expression was increased in people at high cardiovascular risk (Kelly et al., 2016), and correlated with circulating inflammatory IL-1β (Grant et al., 2021), which may be closely associated with inflammatory injury of DKD. However, there was no relation between g*_Tyzzerella* and DKD renal function indicators in this study. The role of g*_Tyzzerella* in DKD progression needs further investigation.

This study reported the relationship between intestinal microecology and DKD progression by associating intestinal microflora with metabolites via multiomics-integrated methods. The results identified the potential role of *g_Abiotrophia*, *g_norank_f_Peptococcaceae*, and *g_Lachnospiraceae_NC2004_Group* in DKD progression, and their involvement in phenylalanine and tryptophan metabolism. These findings offer real promise in finding a new therapeutic strategy that targets protein-bound uremic toxin HA and IAA in DKD. However, our study has some limitations. First, because this was a retrospective study, we lack records of patient drug and dietary intake, so it was not possible to account for the influence that drugs and dietary habits might have had on intestinal flora and the metabolic profile. Second, the sample size was small and would need to be expanded in future studies. Nevertheless, all participants were residents of Guangdong Province, with characteristics and living habits that were relatively concentrated and consistent. Third, the result of gut microbiota is based on 16S rRNA gene sequencing. Further analysis based on gut metagenome, which could provide more bacterial information, is needed.

In conclusion, this study highlights the complex, interactive network of gut microbiota, serum metabolites, and clinical indicators of predialysis DKD patients and provides new insights into the role of gut microbiota and microbiota-related serum metabolites enriched in phenylalanine and tryptophan metabolic pathways in the progression of DKD.

## Data Availability

The original data of 16s rRNA sequencing have been deposited into NCBI databases under the BioProject accession code PRJNA771477 and the BioSample accession number is SAMN22310870.
