# Supplementary material for: The Role of Gut Microbiota and Microbiota-Related Serum Metabolites in the Progression of Diabetic Kidney Disease
Source: Front Pharmacol. 2021 Nov 24;12:757508. doi: 10.3389/fphar.2021.757508 (PMC8652004; doi:10.3389/fphar.2021.757508)
Supplement: Supplementary file 1 [file DataSheet1.docx]

Supplementry Material

**Table of Contents**

[1 Supplementary Figures and Tables 1](#_Toc78374320)

[1.1 Supplementary Figures 1](#_Toc78374321)

[Figure S1. The Rarefaction Curve of Gut microbiota Between Groups in DKD Patients 1](#_Toc78374322)

[Figure S2. Chao and Shannon Indices of Gut microbiota Between Groups in DKD Patients 2](#_Toc78374323)

[Figure S3. Heatmap of 239 Differential Serum Metabolites Associated with 11 Differential Bacteria Between Groups in DKD Patient 4](#_Toc78374324)

[1.2 Supplementary Tables 5](#_Toc78374325)

[Table S1. 239 Differential Serum Metabolites Between Groups in DKD Patients 5](#_Toc78374326)

[Table S2. 192 microbiota -related serum metabolites Between Groups in DKD Patients (*P*＜0.05) 39](#_Toc78374327)

# Supplementary Figures and Tables

## Supplementary Figures

**
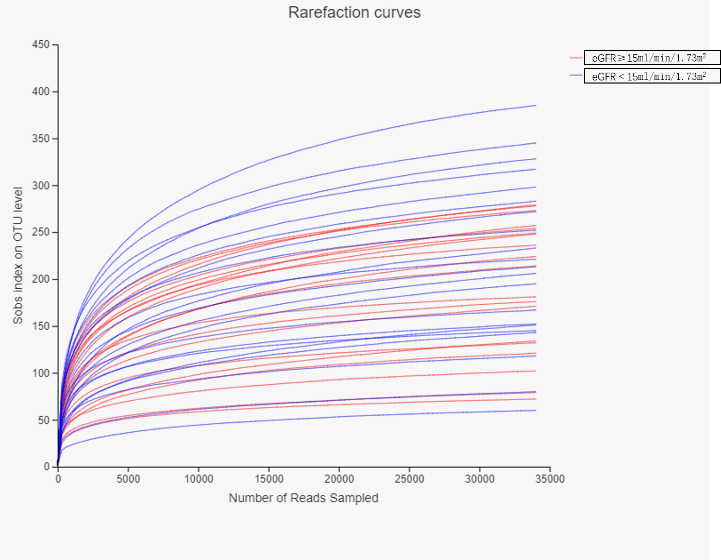
**

### Figure S1. The Rarefaction Curve of Gut microbiota Between Groups in DKD Patients

The horizontal coordinate represents the amount of randomly selected sequencing data and the vertical coordinate represents the Sobs Indices. The red curve indicates DKD non-ESRD group and the blue curve indicates DKD ESRD group.


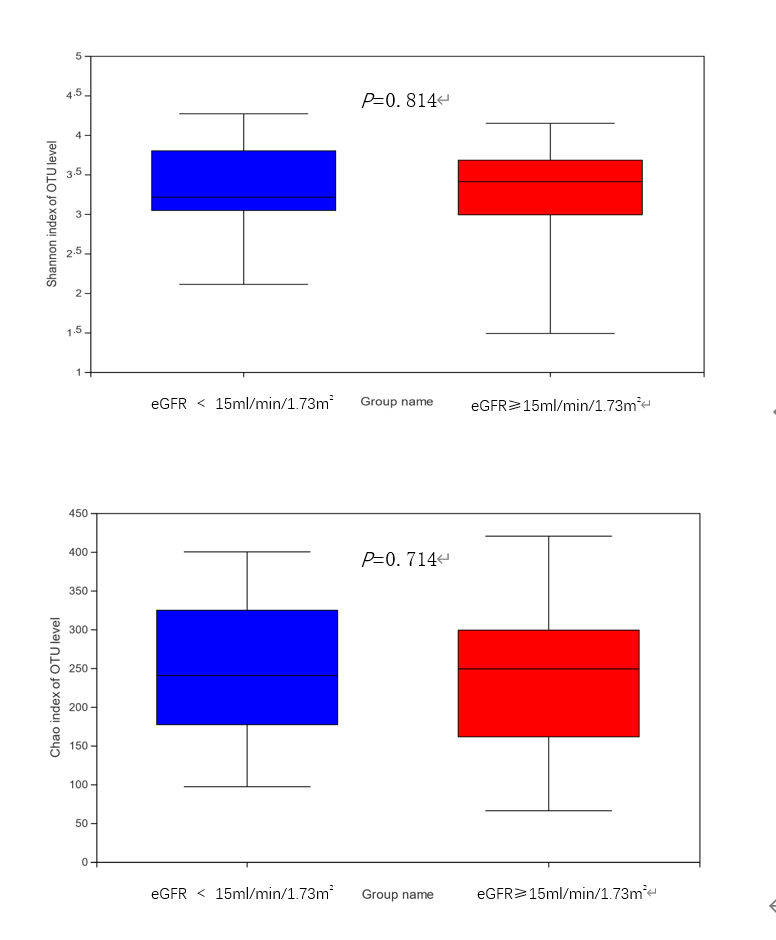


B

A

### Figure S2. Chao and Shannon Indices of Gut microbiota Between Groups in DKD Patients

(A) is Shannon indexes. (B) is Chao indexes.


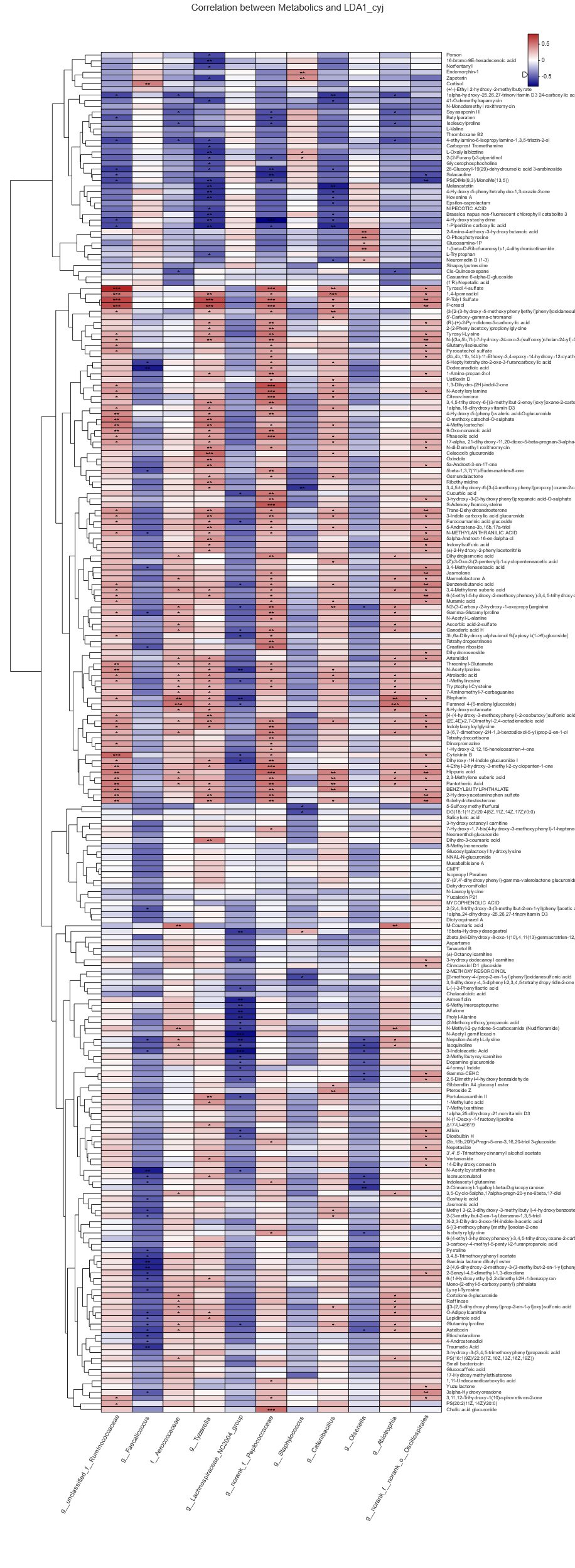


### **Figure S3. Heatmap of 239 Differential Serum Metabolites Associated with 11 Differential** Bacteria Between Groups in DKD Patient

The 239 differential metabolites are listed on the right-hand side of the heatmap, and the 11 differential bacteria are listed on the below in the heatmap. The darker color of the grid indicates high correlation coefficient and the lighter color indicates less correlation coefficient, with red representing a positive correlation and blue representing a negative correlation.

## Supplementary Tables

### Table S1. 239 Differential Serum Metabolites Between Groups in DKD Patients

| Metabolites | Formula | Retention time | M/Z | Library ID | KEGG ID | VIP | P | KEGG Pathway ID | Metabolite changes |
| --- | --- | --- | --- | --- | --- | --- | --- | --- | --- |
| L-Tryptophan | C11H12N2O2 | 2.924583333 | 203.0821968 | HMDB0000929 | C00078 | 1.287538369 | 4.69E-05 | map01100;map04978;map05143;map00260;map01210;map04974;map00380;map01230;map00970;map04726;map00400;map05230 | ↓ |
| Dihydro-3-coumaric acid | C9H10O3 | 4.257533333 | 377.1447712 | - | C11457 | 1.753914789 | 0.0004294 | map00360 | ↑ |
| Traumatic Acid | C12H20O4 | 6.838533333 | 265.10788 | LMFA01170002;HMDB0000933 | C16308 | 1.288824766 | 2.78E-05 | map00592 | ↑ |
| 5-Heptyltetrahydro-2-oxo-3-furancarboxylic acid | C12H20O4 | 6.4909 | 273.1333959 | HMDB0030994 | - | 1.699813148 | 3.53E-05 | - | ↑ |
| L- (-) -3-Phenyllactic acid | C9H10O3 | 4.015383333 | 165.0554466 | - | C05607 | 1.043926652 | 0.012 | map00360 | ↑ |
| Creatine riboside | C9H17N3O6 | 0.693583333 | 244.0936227 | HMDB0240254 | - | 2.259599142 | 5.82E-07 | - | ↑ |
| Raffinose | C18H32O16 | 0.693583333 | 539.1386723 | HMDB0003213 | C00492 | 1.877177541 | 1.02E-05 | map02010;map00052 | ↑ |
| 2-METHOXYRESORCINOL | C7H8O3 | 3.143933333 | 139.0399178 | - | - | 1.616380955 | 0.01484 | - | ↑ |
| N-Acetyl gemifloxacin | C19H20FN5O5 | 4.1007 | 438.0986853 | HMDB0060633 | - | 1.343120596 | 0.009555 | - | ↑ |
| (1'R) -Nepetalic acid | C10H16O3 | 5.58895 | 229.1074781 | HMDB0036117 | - | 1.039712521 | 0.01357 | - | ↓ |
| Dodecanedioic acid | C12H22O4 | 6.4809 | 251.12663 | LMFA01170009;HMDB0000623 | C02678C16308 | 1.561174398 | 2.02E-05 | map00592 | ↑ |
| N- [(3a, 5b, 7b) -7-hydroxy-24-oxo-3- (sulfooxy) cholan-24-yl] -Glycine | C26H43NO8S | 5.719616667 | 574.2688134 | HMDB0002409 | - | 1.314260003 | 0.007363 | - | ↑ |
| 3- (6, 7-dimethoxy-2H-1, 3-benzodioxol-5-yl) prop-2-en-1-ol | C12H14O5 | 4.987483333 | 237.0765133 | HMDB0128659 | - | 1.952322819 | 2.09E-06 | - | ↑ |
| 6-Methylmercaptopurine | C6H6N4S | 0.771566667 | 165.0227056 | HMDB0060412 | C16614 | 1.320697268 | 0.006148 | map00983 | ↑ |
| Furocoumarinic acid glucoside | C17H18O9 | 1.379533333 | 387.0670343 | HMDB0029601 | - | 1.850224275 | 5.69E-05 | - | ↑ |
| 1-Piperidine carboxylic acid | C6H11NO2 | 1.457366667 | 128.0718026 | HMDB0094697 | - | 1.42701749 | 0.003042 | - | ↓ |
| Blepharin | C14H17NO8 | 1.74735 | 326.0875671 | HMDB0029344 | - | 2.073495586 | 4.71E-06 | - | ↑ |
| 2-Hydroxyacetaminophen sulfate | C8H9NO6S | 2.231316667 | 227.9966092 | HMDB0062547 | - | 2.252096322 | 2.04E-05 | - | ↑ |
| Glucocaffeic acid | C15H18O9 | 2.803116667 | 387.0934374 | HMDB0034313 | - | 1.369664079 | 0.001305 | - | ↑ |
| { [3- (2, 5-dihydroxyphenyl) prop-2-en-1-yl] oxy} sulfonic acid | C9H10O6S | 2.864116667 | 291.0179564 | HMDB0134083 | - | 3.128968744 | 2.79E-12 | - | ↑ |
| Furaneol 4- (6-malonylglucoside) | C15H20O11 | 2.90995 | 357.0824728 | HMDB0029778 | - | 2.084261355 | 3.07E-07 | - | ↑ |
| Celecoxib glucuronide | C22H20F3N3O9S | 3.0396 | 540.065973 | HMDB0061132 | - | 1.824500698 | 0.001502 | - | ↑ |
| 1, 3-Dihydro- (2H) -indol-2-one | C8H7NO | 3.106266667 | 178.0509578 | HMDB0061918 | C12312 | 1.336215322 | 0.0006881 | - | ↑ |
| Indoxylsulfuric acid | C8H7NO4S | 3.234416667 | 212.0019865 | - | - | 1.86006495 | 1.92E-06 | - | ↑ |
| Soyasaponin III | C42H68O14 | 3.368083333 | 817.4425582 | HMDB0034651 | C19865 | 1.827652785 | 2.30E-05 | - | ↓ |
| 28-Glucosyl-19 (29) -dehydroursolic acid 3-arabinoside | C41H64O12 | 3.51475 | 729.421896 | HMDB0038019 | - | 2.08054707 | 2.87E-06 | - | ↓ |
| P-Tolyl Sulfate | C7H8O4S | 3.7234 | 187.0068756 | - | - | 1.496249558 | 0.000183 | - | ↑ |
| 3-hydroxy-3- (3-hydroxyphenyl) propanoic acid-O-sulphate | C9H10O7S | 4.1102 | 282.9910222 | HMDB0059967 | - | 1.583670074 | 0.0006161 | - | ↑ |
| 6- (4-ethyl-5-hydroxy-2-methoxyphenoxy) -3, 4, 5-trihydroxyoxane-2-carboxylic acid | C15H20O9 | 4.1102 | 325.0922597 | HMDB0128044 | - | 2.509328771 | 1.66E-08 | - | ↑ |
| 5beta-1, 3, 7 (11) -Eudesmatrien-8-one | C15H20O | 4.306683333 | 237.1240328 | HMDB0035965 | - | 2.046190136 | 3.41E-06 | - | ↑ |
| 3, 4, 5-trihydroxy-6- [3- (4-methoxyphenyl) propoxy] oxane-2-carboxylic acid | C16H22O8 | 4.54035 | 341.1219773 | HMDB0135749 | - | 1.785297611 | 0.0009302 | - | ↑ |
| 3, 6-dihydroxy-4, 5-diphenyl-2, 3, 4, 5-tetrahydropyridin-2-one | C17H15NO3 | 5.128466667 | 326.1030476 | HMDB0134800 | - | 1.415198949 | 0.01441 | - | ↑ |
| Garcinia lactone dibutyl ester | C14H22O7 | 5.315633333 | 283.1182263 | HMDB0040462 | - | 1.892000699 | 2.23E-08 | - | ↑ |
| 6- (1-Hydroxyethyl) -2, 2-dimethyl-2H-1-benzopyran | C13H16O2 | 5.518616667 | 249.1128415 | HMDB0031553 | - | 2.149952374 | 1.04E-06 | - | ↑ |
| Porson | C22H26O6 | 5.58895 | 423.1191738 | HMDB0030810 | - | 1.49595343 | 0.0004631 | - | ↓ |
| 2-Benzyl-4, 5-dimethyl-1, 3-dioxolane | C12H16O2 | 5.728933333 | 237.1128491 | HMDB0039841 | - | 1.645355075 | 2.96E-07 | - | ↑ |
| Sinapoylputrescine | C15H22N2O4 | 5.8376 | 275.1426607 | HMDB0033464 | - | 1.11126202 | 0.008992 | - | ↓ |
| 8-Hydroxyoctanoate | C8H16O3 | 6.038583333 | 159.102303 | HMDB0061914 | - | 2.202179577 | 8.70E-08 | - | ↑ |
| N-Lauroylglycine | C14H27NO3 | 6.057916667 | 294.1453459 | HMDB0013272 | - | 1.365880046 | 0.003539 | - | ↑ |
| 8-Methylnonenoate | C10H18O2 | 6.082433333 | 151.1124344 | LMFA01030994;HMDB0012183 | C18202 | 1.314355468 | 0.003055 | map01100;map00360 | ↑ |
| Dehydrovomifoliol | C13H18O3 | 6.229066667 | 267.1229643 | HMDB0036819;LMPR0103050009 | C04223C02533 | 1.543690676 | 0.0006836 | - | ↑ |
| (3b, 16b, 20R) -Pregn-5-ene-3, 16, 20-triol 3-glucoside | C27H44O8 | 6.573216667 | 541.3020369 | HMDB0041331 | - | 1.519736622 | 0.001723 | - | ↑ |
| Carboprost Tromethamine | C21H36O5 | 7.80865 | 367.2481327 | HMDB0014573 | - | 1.049210187 | 0.002224 | - | ↓ |
| PS (20:2 (11Z, 14Z) /20:0) | C46H86NO10P | 10.32316667 | 888.5983992 | LMGP03010577HMDB0112578 | - | 1.064845187 | 0.01072 | - | ↑ |
| Hovenine A | C27H42N4O4 | 9.218733333 | 531.3204584 | HMDB0030200 | - | 1.045997616 | 0.02926 | - | ↓ |
| Goshuyic acid | C14H24O2 | 6.660383333 | 269.1751333 | LMFA01030257;HMDB0000560 | - | 1.260588996 | 0.0001431 | - | ↑ |
| Jasmonic acid | C12H18O3 | 6.59255 | 209.1179156 | HMDB0032797;LMFA02020001 | C08491 | 2.042397832 | 0.0001194 | map01100;map00592 | ↑ |
| 1, 11-Undecanedicarboxylic acid | C13H24O4 | 6.529716667 | 243.1596655 | HMDB0002327 | - | 1.003077714 | 0.02618 | - | ↑ |
| (3b, 4b, 11b, 14b) -11-Ethoxy-3, 4-epoxy-14-hydroxy-12-cyathen-15-al 14-xyloside | C27H42O8 | 6.4809 | 539.2846528 | HMDB0034617 | - | 1.173284678 | 0.03816 | - | ↑ |
| Armexifolin | C15H18O4 | 6.258066667 | 283.0966556 | HMDB0035596 | - | 1.263172596 | 0.04326 | - | ↑ |
| Zapoterin | C26H30O8 | 6.248233333 | 491.1710385 | HMDB0035984 | C08788 | 2.034484148 | 0.0001649 | - | ↓ |
| Lysyl-Tyrosine | C15H23N3O4 | 6.229066667 | 308.1608446 | HMDB0028963 | - | 1.946756838 | 7.11E-07 | - | ↑ |
| Neomenthol-glucuronide | C16H28O7 | 6.163083333 | 331.1752406 | HMDB0060012 | - | 1.963595392 | 8.81E-05 | - | ↑ |
| 3alpha-Hydroxyoreadone | C14H20O4 | 5.9361 | 251.1281706 | HMDB0036047 | - | 1.359504675 | 0.0002526 | - | ↑ |
| Cinncassiol D1 glucoside | C26H42O10 | 5.895933333 | 535.2532003 | HMDB0034677 | - | 1.257408276 | 0.01158 | - | ↑ |
| Ustiloxin D | C23H34N4O8 | 5.49895 | 475.2186711 | HMDB0041054 | - | 1.928544504 | 0.0001122 | - | ↑ |
| Jasmolone | C11H16O2 | 5.49895 | 225.1125994 | HMDB0030039 | - | 1.66345312 | 6.43E-05 | - | ↑ |
| Endomorphin-1 | C34H38N6O5 | 5.45895 | 631.2698201 | HMDB0005773 | C15890 | 1.679092178 | 0.0007001 | map04080 | ↓ |
| Dihydrojasmonic acid | C12H20O3 | 5.418616667 | 257.1388022 | HMDB0033601 | - | 1.429334218 | 0.0006703 | - | ↑ |
| Asteltoxin | C23H30O7 | 5.369133333 | 417.1909939 | HMDB0029464 | - | 2.011068748 | 9.05E-08 | - | ↑ |
| Diosbulbin H | C23H30O7 | 5.326466667 | 399.1836508 | HMDB0036781 | - | 1.404351693 | 0.005797 | - | ↑ |
| Cucurbic acid | C12H20O3 | 5.315633333 | 257.1389922 | HMDB0029388 | C08482 | 1.105971152 | 0.009375 | - | ↑ |
| Brassica napus non-fluorescent chlorophyll catabolite 3 | C34H38N4O8 | 5.2063 | 651.2424512 | HMDB0040917 | - | 1.265323633 | 0.01033 | - | ↓ |
| N-di-Demethyl roxithromycin | C40H74N2O14 | 5.05915 | 843.4555332 | HMDB0060612 | - | 1.990379733 | 6.48E-06 | - | ↑ |
| Marmelolactone A | C10H14O2 | 5.05915 | 211.0972127 | HMDB0036042 | - | 1.85641948 | 2.73E-06 | - | ↑ |
| Ganoderic acid H | C32H44O9 | 5.05915 | 609.2527453 | HMDB0035987 | - | 1.421131836 | 4.16E-07 | - | ↑ |
| Artemidiol | C13H14O4 | 5.04965 | 279.0863391 | HMDB0030648 | - | 1.754322477 | 0.0002354 | - | ↑ |
| Phaseolic acid | C12H22O6 | 4.90065 | 243.1231976 | LMFA01060200;HMDB0031897 | C10483 | 1.879847498 | 4.40E-07 | - | ↑ |
| Norfentanyl | C14H20N2O | 4.880816667 | 253.1339998 | HMDB0061006 | - | 1.135685733 | 0.005124 | - | ↓ |
| 9-Oxo-nonanoic acid | C9H16O3 | 4.871333333 | 217.1076525 | LMFA01060160;HMDB0094711 | - | 1.801083353 | 1.51E-07 | - | ↑ |
| N-METHYLANTHRANILIC ACID | C8H9NO2 | 4.822333333 | 150.0558643 | - | C03005 | 2.537078328 | 1.54E-10 | - | ↑ |
| PS (16:1 (9Z) /22:5 (7Z, 10Z, 13Z, 16Z, 19Z) ) | C44H74NO10P | 4.770333333 | 828.4799639 | HMDB0112369 | - | 1.944642284 | 1.22E-05 | - | ↑ |
| Cortolone-3-glucuronide | C27H42O11 | 4.761 | 541.2652937 | HMDB0010320 | - | 2.107279711 | 1.61E-11 | - | ↑ |
| Cortisol | C21H30O5 | 4.648 | 407.2047677 | LMST02030001;HMDB0000063 | C00735 | 1.141907254 | 0.0009366 | map01100;map05215;map05200;map04976;map04960;map04080;map00140 | ↓ |
| 3, 4-Methylene suberic acid | C10H14O4 | 4.6 | 197.081637 | HMDB0059768 | - | 2.164620625 | 3.25E-07 | - | ↑ |
| Melanostatin | C13H24N4O3 | 4.6 | 321.1348234 | HMDB0005764 | - | 1.382915197 | 0.0008422 | - | ↓ |
| {3- [2- (3-hydroxy-5-methoxyphenyl) ethyl] phenyl} oxidanesulfonic acid | C15H16O6S | 4.55035 | 323.0586265 | HMDB0129925 | - | 1.47258769 | 0.004156 | - | ↑ |
| O-Phosphotyrosine | C9H12NO6P | 4.48235 | 296.0112669 | HMDB0006049 | C06501 | 1.131526468 | 0.001592 | - | ↓ |
| Indole-3 acetic acid | C10H9NO2 | 4.47285 | 174.0557968 | - | C00954 | 1.175620734 | 0.0001708 | map01100;map00380 | ↑ |
| Tyrosol 4-sulfate | C8H10O5S | 4.326016667 | 217.0167997 | HMDB0041785 | - | 1.042519915 | 0.0377 | - | ↑ |
| Glucosamine-1P | C6H14NO8P | 4.267366667 | 294.013821 | HMDB0001109 | - | 1.675704727 | 0.0004328 | - | ↓ |
| 2, 3-Methylene suberic acid | C10H14O4 | 4.229033333 | 197.0814293 | HMDB0059779 | - | 1.969586046 | 6.30E-06 | - | ↑ |
| Indolylacryloylglycine | C13H12N2O3 | 4.139866667 | 243.077058 | HMDB0006005 | - | 2.329921916 | 1.73E-06 | - | ↑ |
| 4-Ethyl-2-hydroxy-3-methyl-2-cyclopenten-1-one | C8H12O2 | 4.090866667 | 185.0812459 | HMDB0039673 | - | 1.737837126 | 6.66E-05 | - | ↑ |
| 4-Hydroxy-5- (phenyl) -valeric acid-O-glucuronide | C17H22O9 | 4.043533333 | 369.1183795 | HMDB0059980 | - | 1.904426636 | 0.0006834 | - | ↑ |
| [2-methoxy-4- (prop-2-en-1-yl) phenyl] oxidanesulfonic acid | C10H12O5S | 3.943883333 | 289.0386277 | HMDB0135245 | - | 1.723245651 | 0.0001888 | - | ↑ |
| Trans-3-Hydroxycinnamate | C9H8O3 | 3.943883333 | 209.0453966 | HMDB0001713 | C12621 | 1.166086195 | 0.01235 | map00360 | ↑ |
| 4-Hydroxystachydrine | C7H13NO3 | 3.7234 | 158.0818784 | HMDB0029230 | C08269 | 1.381764108 | 0.003069 | - | ↓ |
| P-cresol | C7H8O | 3.7234 | 107.0510463 | HMDB0001858 | C01468 | 1.586032462 | 0.0001231 | map01100;map04974 | ↑ |
| 6- (4-ethyl-3-hydroxyphenoxy) -3, 4, 5-trihydroxyoxane-2-carboxylic acid | C14H18O8 | 3.5781 | 313.0926423 | HMDB0126441 | - | 1.844800224 | 0.001132 | - | ↑ |
| O-methoxycatechol-O-sulphate | C7H8O5S | 3.5781 | 203.0015571 | HMDB0060013 | - | 1.830345341 | 0.0002096 | - | ↑ |
| 4-Methylcatechol | C7H8O2 | 3.5729 | 123.045371 | HMDB0000873 | C06730 | 2.027581033 | 0.0001318 | map01100 | ↑ |
| 4-Hydroxy-5-phenyltetrahydro-1, 3-oxazin-2-one | C10H11NO3 | 3.5729 | 192.065939 | HMDB0060389 | C16595 | 1.239537301 | 0.01044 | map00982 | ↓ |
| 3-Indole carboxylic acid glucuronide | C15H15NO8 | 3.563566667 | 336.0715041 | HMDB0013189 | - | 2.868175005 | 4.99E-09 | - | ↑ |
| PS (DiMe (9, 3) /MonoMe (13, 5) ) | C47H80NO12P | 3.544233333 | 926.5371038 | HMDB0061586 | - | 1.239301386 | 2.46E-05 | - | ↓ |
| 41-O-demethylrapamycin | C50H77NO13 | 3.524733333 | 880.5224622 | HMDB0061048 | - | 1.245983028 | 0.0008409 | - | ↓ |
| Solacauline | C43H69NO14 | 3.44675 | 804.4561126 | HMDB0029373 | - | 1.481669812 | 7.86E-07 | - | ↓ |
| [4- (4-hydroxy-3-methoxyphenyl) -2-oxobutoxy] sulfonic acid | C11H14O7S | 3.436916667 | 289.0380674 | HMDB0135712 | - | 2.512964446 | 5.19E-08 | - | ↑ |
| 2- (2-Phenylacetoxy) propionylglycine | C13H15NO5 | 3.427583333 | 264.0873206 | HMDB0059732 | - | 1.570378488 | 0.003658 | - | ↑ |
| Epsilon-caprolactam | C6H11NO | 3.40775 | 158.0820839 | HMDB0062769 | C06593 | 1.039447328 | 0.007254 | map01100 | ↓ |
| Dopamine glucuronide | C14H19NO8 | 3.397416667 | 350.0875181 | HMDB0010329 | - | 2.509797183 | 2.61E-05 | - | ↑ |
| N-Acetylarylamine | C8H9NO | 3.329416667 | 134.0612268 | HMDB0001250 | C07565 | 2.08728103 | 8.46E-06 | - | ↑ |
| (±) -2-Hydroxy-2-phenylacetonitrile | C8H7NO | 3.234416667 | 132.045438 | HMDB0034666 | - | 2.70190582 | 1.43E-07 | - | ↑ |
| Pyrocatechol sulfate | C6H6O5S | 2.962766667 | 188.9858377 | HMDB0059724 | - | 1.242539452 | 0.002072 | - | ↑ |
| 2-Amino-4-ethoxy-3-hydroxybutanoic acid | C6H13NO4 | 2.924583333 | 144.0666158 | HMDB0032862 | - | 1.00594557 | 0.02526 | - | ↓ |
| 5-Sulfoxymethylfurfural | C6H6O6S | 2.894783333 | 204.9807438 | HMDB0059752 | - | 1.224408648 | 0.01488 | - | ↑ |
| Portulacaxanthin II | C18H18N2O7 | 2.68845 | 355.0944125 | HMDB0012281 | C08565 | 1.439131691 | 0.001199 | - | ↑ |
| Gamma-Glutamylproline | C10H16N2O5 | 2.587466667 | 225.0874242 | HMDB0029157 | - | 1.577296822 | 9.30E-07 | - | ↑ |
| Lepidimoic acid | C12H18O10 | 2.577133333 | 343.0663347 | HMDB0041096 | - | 2.629870846 | 3.45E-08 | - | ↑ |
| Salicyluric acid | C9H9NO4 | 1.957666667 | 194.0456631 | HMDB0000840 | C07588 | 1.671326459 | 0.00237 | - | ↑ |
| Glutaminylproline | C10H17N3O4 | 1.651683333 | 288.1195225 | HMDB0028805 | - | 1.724308298 | 2.87E-07 | - | ↑ |
| Dihyroxy-1H-indole glucuronide I | C14H15NO8 | 1.389033333 | 324.071775 | HMDB0059997 | - | 1.580411155 | 0.002899 | - | ↑ |
| (2-Methoxyethoxy) propanoic acid | C6H12O4 | 1.344866667 | 147.0663622 | HMDB0059705 | - | 1.576249491 | 0.0002556 | - | ↑ |
| N-Acetyl-L-alanine | C5H9NO3 | 1.267033333 | 130.0511511 | HMDB0000766 | - | 1.000955856 | 1.70E-06 | - | ↑ |
| Isobutyrylglycine | C6H11NO3 | 0.878566667 | 144.066976 | HMDB0000730 | - | 1.857355537 | 4.65E-05 | - | ↑ |
| L-Oxalylalbizziine | C6H9N3O6 | 0.819733333 | 200.0293837 | HMDB0039164 | - | 1.015901734 | 0.0001919 | - | ↓ |
| Ascorbic acid-2-sulfate | C6H8O9S | 0.800066667 | 254.9809565 | HMDB0060649 | - | 1.545720918 | 1.44E-07 | - | ↑ |
| Alfalone | C17H14O5 | 0.771566667 | 333.0510792 | LMPK12050105;HMDB0038811 | - | 1.325104741 | 0.0006396 | - | ↑ |
| MYCOPHENOLIC ACID | C17H20O6 | 5.7929 | 321.1325518 | HMDB0015159 | C20380 | 1.498910516 | 0.02995 | - | ↑ |
| CMPF | C12H16O5 | 6.078383333 | 223.096045 | - | - | 1.746911877 | 0.0003283 | - | ↑ |
| 17-alpha, 21-dihydroxy-11, 20-dioxo-5-beta-pregnan-3-alpha-yl-beta-d-glucuronide | C27H40O11 | 4.90695 | 558.2908232 | - | - | 2.595539262 | 2.95E-11 | - | ↑ |
| Hippuric acid | C9H9NO3 | 3.3362 | 180.0648553 | HMDB0000714 | C01586 | 2.307636577 | 2.28E-06 | map00360 | ↑ |
| Ribothymidine | C10H14N2O6 | 0.70035 | 259.092221 | HMDB0000884 | - | 2.198658126 | 2.64E-07 | - | ↑ |
| (2E, 4E) -2, 7-Dimethyl-2, 4-octadienedioic acid | C10H14O4 | 4.22965 | 181.0851327 | HMDB0034099 | - | 3.360952504 | 1.15E-06 | - | ↑ |
| 3, 4, 5-Trimethoxyphenyl acetate | C11H14O5 | 5.545733333 | 209.0803749 | HMDB0031722 | - | 3.397133502 | 1.53E-08 | - | ↑ |
| 14-Dihydroxycornestin | C16H20O6 | 5.680133333 | 309.132561 | - | C08483 | 1.633950201 | 0.001064 | - | ↑ |
| Mono- (2-ethyl-5-carboxypentyl) phthalate | C16H20O6 | 6.02505 | 331.1140839 | HMDB0094647 | - | 3.528334523 | 3.91E-06 | - | ↑ |
| Methyl 3- (2, 3-dihydroxy-3-methylbutyl) -4-hydroxybenzoate | C13H18O5 | 6.587516667 | 237.1113932 | HMDB0032796 | - | 3.073886571 | 1.25E-06 | - | ↑ |
| Gamma-CEHC | C15H20O4 | 5.5909 | 265.1428855 | HMDB0001931 | - | 1.641759245 | 0.0002224 | - | ↑ |
| 3, 4-Methylenesebacic acid | C12H18O4 | 5.408083333 | 209.1166785 | HMDB0059729 | - | 2.03781033 | 4.86E-05 | - | ↑ |
| 2- [2, 4, 6-trihydroxy-3- (3-methylbut-2-en-1-yl) phenyl] acetic acid | C13H16O5 | 5.077766667 | 235.0959389 | HMDB0125460 | - | 1.8727037 | 0.00232 | - | ↑ |
| 1alpha, 18-dihydroxyvitamin D3 | C27H44O3 | 6.347033333 | 399.3248792 | LMST03020254 | - | 1.94006365 | 0.008815 | - | ↑ |
| BENZYLBUTYLPHTHALATE | C19H20O4 | 7.72945 | 313.1429293 | - | - | 1.010159523 | 0.008186 | - | ↑ |
| 15beta-Hydroxydesogestrel | C22H30O2 | 6.2517 | 327.2307889 | HMDB0060707 | - | 1.145843235 | 0.04603 | - | ↓ |
| 1alpha, 25-dihydroxy-21-norvitamin D3 | C26H42O3 | 6.241533333 | 403.3199425 | LMST03020048 | - | 1.248075025 | 0.003291 | - | ↑ |
| Glucosylgalactosyl hydroxylysine | C18H34N2O13 | 6.230533333 | 469.2059786 | HMDB0000585 | - | 2.254404135 | 5.33E-05 | - | ↑ |
| Etiocholanolone | C19H30O2 | 6.1572 | 273.2207249 | HMDB0000490 | C04373 | 3.06264893 | 2.64E-06 | map00140 | ↑ |
| Cholic acid glucuronide | C30H48O11 | 5.139266667 | 602.3557731 | LMST05010044;HMDB0002577 | - | 1.619301199 | 0.04916 | - | ↑ |
| 17-U-46619 | C21H32O4 | 5.22375 | 331.226369 | - | - | 1.892689971 | 5.40E-05 | - | ↑ |
| 5a-Androst-3-en-17-one | C19H28O | 5.181766667 | 273.2206777 | HMDB0006046;LMST02020094 | - | 4.351498562 | 1.10E-09 | - | ↑ |
| Tetrahydrocortisone | C21H32O5 | 4.735616667 | 347.2215671 | HMDB0000903 | C05470 | 2.031883498 | 6.06E-08 | map00140 | ↑ |
| Tetrahydrogestrinone | C21H28O2 | 4.596633333 | 313.2160617 | HMDB0004626 | - | 2.845398067 | 2.94E-09 | - | ↑ |
| Xi-2, 3-Dihydro-2-oxo-1H-indole-3-acetic acid | C10H9NO3 | 2.01995 | 209.0915976 | HMDB0035514 | - | 1.119106004 | 0.0006946 | - | ↑ |
| Glycerophosphocholine | C8H20NO6P | 0.630366667 | 280.0919922 | HMDB0000086 | C00670 | 1.149957284 | 0.0005978 | map00565;map00564;map05231 | ↓ |
| N- (1-Deoxy-1-fructosyl) proline | C11H19NO7 | 0.729183333 | 278.1232053 | HMDB0038493 | - | 1.78350489 | 2.28E-06 | - | ↑ |
| L-Valine | C5H11NO2 | 0.758683333 | 235.1647002 | HMDB0000883 | C00183 | 1.215772978 | 0.02168 | map01100;map04978;map00770;map00290;map01210;map02010;map01230;map04974;map00970;map00280;map05230 | ↓ |
| Osmundalactone | C6H8O3 | 0.895166667 | 146.0806657 | HMDB0031303 | - | 1.79464122 | 2.69E-06 | - | ↑ |
| 4-ethylamino-6-isopropylamino-1, 3, 5-triazin-2-ol | C8H15N5O | 0.974333333 | 239.161309 | HMDB0062766 | C06552 | 1.203781624 | 1.58E-06 | map01100 | ↓ |
| Prolyl-Alanine | C8H14N2O3 | 1.17915 | 169.0971715 | HMDB0029010 | - | 1.60975935 | 0.01861 | - | ↑ |
| Dinorpromazine | C15H16N2S | 1.372983333 | 257.1130164 | - | - | 1.694990153 | 1.48E-06 | - | ↑ |
| NIPECOTIC ACID | C6H11NO2 | 1.460466667 | 130.0859522 | - | - | 1.364502097 | 0.009615 | - | ↓ |
| 1-Methyluric acid | C6H6N4O3 | 1.5863 | 183.0507062 | HMDB0003099 | C16359 | 3.102684965 | 4.94E-05 | map00232 | ↑ |
| Allixin | C12H18O4 | 1.620966667 | 244.1537544 | HMDB0040705 | - | 2.249564876 | 0.0001544 | - | ↑ |
| N-Acetylcystathionine | C9H16N2O5S | 2.375266667 | 247.0741598 | HMDB0002381 | - | 2.855506388 | 0.000127 | - | ↑ |
| Muramic acid | C9H17NO7 | 2.7324 | 234.096338 | HMDB0003254 | C06470 | 2.882561614 | 1.32E-09 | - | ↑ |
| 2-Cinnamoyl-1-galloyl-beta-D-glucopyranose | C22H22O11 | 2.7519 | 480.1531605 | HMDB0039189 | - | 2.722124802 | 0.0001113 | - | ↑ |
| 7-Aminomethyl-7-carbaguanine | C7H9N5O | 2.761566667 | 180.0873941 | HMDB0011690 | C16675 | 2.741799452 | 1.79E-09 | map01100;map00790 | ↑ |
| Butylparaben | C11H14O3 | 2.801233333 | 212.1279001 | HMDB0032575 | - | 1.341263786 | 0.005987 | - | ↓ |
| Aspartame | C14H18N2O5 | 2.8524 | 317.1136577 | HMDB0001894 | C11045 | 2.299901282 | 0.015 | map04742 | ↑ |
| 1- (beta-D-Ribofuranosyl) -1, 4-dihydronicotinamide | C11H16N2O5 | 2.8824 | 221.0910856 | HMDB0011648 | C15497 | 1.36588685 | 0.008102 | - | ↓ |
| Cytokinin B | C12H11N5 | 2.8824 | 190.0893083 | HMDB0039238 | C11263 | 2.192170284 | 0.0001028 | - | ↑ |
| Glutamylisoleucine | C11H20N2O5 | 3.195216667 | 243.1336871 | HMDB0028822 | - | 1.519523625 | 0.02893 | - | ↑ |
| Indoleacetyl glutamine | C15H17N3O4 | 3.220033333 | 304.1288746 | HMDB0013240 | - | 3.139326737 | 4.44E-05 | - | ↑ |
| N-Monodemethyl roxithromycin | C39H72N2O15 | 3.3167 | 405.2543613 | HMDB0060845 | - | 1.665671859 | 0.005004 | - | ↓ |
| Verbasoside | C20H30O12 | 4.209816667 | 504.2069678 | HMDB0039233 | - | 2.097769266 | 0.0006578 | - | ↑ |
| Thromboxane B2 | C20H34O6 | 4.323316667 | 782.5059142 | HMDB0003252;LMFA03030002 | C05963 | 1.291788532 | 0.006625 | map00590;map01100;map04976;map04726 | ↓ |
| 3, 5-Cyclo-5alpha, 17alpha-pregn-20-yne-6beta, 17-diol | C21H30O2 | 4.638633333 | 315.2313138 | - | C15468 | 3.463927154 | 6.32E-10 | - | ↑ |
| 3-hydroxyoctanoyl carnitine | C15H29NO5 | 4.76545 | 326.1957431 | HMDB0061634 | - | 2.518300536 | 8.68E-06 | - | ↑ |
| Benzenebutanoic acid | C10H12O2 | 4.82445 | 165.0909942 | HMDB0000543 | - | 3.371173755 | 2.33E-08 | - | ↑ |
| 5-Androstene-3b, 16b, 17a-triol | C19H30O3 | 5.128433333 | 307.2260112 | LMST02020098;HMDB0000523 | - | 4.066484932 | 8.31E-11 | - | ↑ |
| 5- [(3-methoxyphenyl) methyl] oxolan-2-one | C12H14O3 | 5.408083333 | 239.1271872 | HMDB0127763 | - | 1.981900832 | 0.001192 | - | ↑ |
| 3', 4', 5'-Trimethoxycinnamyl alcohol acetate | C14H18O5 | 5.535916667 | 267.1216364 | HMDB0040891 | - | 2.806468203 | 4.55E-05 | - | ↑ |
| 2, 6-Dimethyl-4-hydroxybenzaldehyde | C9H10O2 | 5.5909 | 151.0749553 | - | - | 1.514349161 | 0.0004314 | - | ↑ |
| Musabalbisiane A | C23H28O12 | 6.078383333 | 535.1244656 | HMDB0038680 | - | 2.483869829 | 0.000443 | - | ↑ |
| Cholacalcioic acid | C24H36O3 | 6.335866667 | 373.272508 | - | - | 1.299870077 | 0.02078 | - | ↑ |
| Yucalexin P21 | C20H32O2 | 6.335866667 | 287.2359441 | HMDB0036754 | - | 1.530671017 | 0.02096 | - | ↑ |
| Neuromedin B (1-3) | C12H22N4O5 | 9.7415 | 622.3569475 | HMDB0013016 | - | 1.577145373 | 0.01571 | - | ↓ |
| DG (18:1 (11Z) /20:4 (8Z, 11Z, 14Z, 17Z) /0:0) | C41H70O5 | 12.25135 | 660.5548149 | HMDB0007200 | - | 1.148024423 | 0.01843 | - | ↑ |
| 1alpha, 24-dihydroxy-25, 26, 27-trinorvitamin D3 | C24H38O3 | 7.2668 | 375.2882426 | LMST03020023 | - | 1.837387785 | 0.0004587 | - | ↑ |
| 1alpha-hydroxy-25, 26, 27-trinorvitamin D3 24-carboxylic acid | C24H36O4 | 6.9135 | 389.2677334 | LMST03020022 | - | 1.223362192 | 0.006027 | - | ↓ |
| 1-Hydroxy-2, 12, 15-heneicosatrien-4-one | C21H36O2 | 6.671833333 | 285.2571428 | LMFA05000600;HMDB0036741 | - | 2.403821677 | 3.44E-06 | - | ↑ |
| 2- (3-methylbut-2-en-1-yl) benzene-1, 3, 5-triol | C11H14O3 | 6.597516667 | 195.1009573 | HMDB0124918 | - | 2.763886035 | 8.33E-08 | - | ↑ |
| Yuzu lactone | C12H20O2 | 6.3682 | 219.1366249 | HMDB0032901 | - | 1.333513397 | 0.006148 | - | ↑ |
| 5alpha-Androst-16-en-3alpha-ol | C19H30O | 6.2837 | 257.2259399 | - | - | 2.610703461 | 1.02E-06 | - | ↑ |
| NNAL-N-glucuronide | C16H24N3O8+ | 6.230533333 | 409.1484737 | HMDB0060498 | C19606 | 2.00677056 | 6.30E-05 | map05204;map00980 | ↑ |
| 4-Androstenediol | C19H30O2 | 6.1572 | 255.2099961 | LMST02020105;HMDB0005849 | C14210 | 2.764645128 | 1.05E-05 | - | ↑ |
| 3-carboxy-4-methyl-5-pentyl-2-furanpropanoic acid | C14H20O5 | 6.087866667 | 251.1268607 | HMDB0061643 | - | 2.822488404 | 4.20E-06 | - | ↑ |
| Isopeopyl Paraben | C10H12O3 | 6.078383333 | 181.0855157 | - | - | 1.695101123 | 0.0002369 | - | ↑ |
| 3-hydroxydodecanoyl carnitine | C19H37NO5 | 6.036216667 | 342.2635687 | HMDB0061638 | - | 1.566828022 | 0.006784 | - | ↑ |
| 3-hydroxy-3- (3, 4, 5-trimethoxyphenyl) propanoic acid | C12H16O6 | 5.8559 | 221.0800922 | HMDB0141328 | - | 2.901547845 | 6.02E-08 | - | ↑ |
| Dictyoquinazol A | C17H16N2O4 | 5.7199 | 330.1438341 | HMDB0040435 | - | 2.078419606 | 0.0002298 | - | ↑ |
| Gibberellin A4 glucosyl ester | C25H34O10 | 5.6744 | 495.2219485 | HMDB0038610 | - | 1.99182028 | 0.004068 | - | ↑ |
| 5'-Carboxy-gamma-chromanol | C18H26O4 | 5.6649 | 307.1892973 | HMDB0012799 | - | 1.540358259 | 0.02465 | - | ↑ |
| Cis-Quinceoxepane | C12H20O | 5.439083333 | 198.1844176 | HMDB0038108 | - | 1.084359906 | 0.03471 | - | ↓ |
| 3, 11, 12-Trihydroxy-1 (10) -spirovetiven-2-one | C15H24O4 | 5.408083333 | 269.1741429 | HMDB0038154 | - | 1.338713972 | 0.007877 | - | ↑ |
| Isoquinoline | C9H7N | 5.325583333 | 130.0638867 | HMDB0034244 | C06323 | 2.609023868 | 2.93E-05 | - | ↑ |
| 2- [4, 6-dihydroxy-2-methoxy-3- (3-methylbut-2-en-1-yl) phenyl] acetic acid | C14H18O5 | 5.315583333 | 267.1220786 | HMDB0125455 | - | 2.901955095 | 2.08E-09 | - | ↑ |
| 5'- (3', 4'-dihydroxyphenyl) -gamma-valerolactone glucuronide | C18H22O10 | 5.306083333 | 399.1277911 | - | - | 1.817130324 | 0.006615 | - | ↑ |
| Tanacetol B | C17H28O4 | 5.295916667 | 314.2323742 | HMDB0035075 | - | 1.736905675 | 0.0001997 | - | ↑ |
| 6-dehydrotestosterone | C19H26O2 | 5.212933333 | 287.2000913 | LMST02020082 | - | 3.841519982 | 9.44E-07 | - | ↑ |
| (Z) -3-Oxo-2- (2-pentenyl) -1-cyclopenteneacetic acid | C12H16O3 | 5.160433333 | 209.1162626 | HMDB0030197 | - | 2.304982855 | 3.65E-05 | - | ↑ |
| Trans-Dehydroandrosterone | C19H28O2 | 5.128433333 | 289.2158492 | - | C01227 | 4.281991144 | 6.48E-09 | map01100;map05215;map00140;map04913;map05200 | ↑ |
| 17-Hydroxymethylethisterone | C22H30O3 | 4.948766667 | 365.206814 | HMDB0060710 | - | 2.624523618 | 2.25E-05 | - | ↑ |
| 7-Hydroxy-1, 7-bis (4-hydroxy-3-methoxyphenyl) -1-heptene-3, 5-dione | C21H22O7 | 4.89695 | 387.1460369 | HMDB0040355 | - | 1.46719548 | 0.02499 | - | ↑ |
| 2beta, 9xi-Dihydroxy-8-oxo-1 (10) , 4, 11 (13) -germacratrien-12, 6alpha-olide | C15H18O5 | 4.85545 | 279.1223879 | HMDB0036662 | - | 1.37326329 | 0.01298 | - | ↑ |
| Nepetaside | C16H26O8 | 4.82445 | 311.1483468 | HMDB0038149 | - | 1.478144658 | 0.01401 | - | ↑ |
| 16-bromo-9E-hexadecenoic acid | C16H29BrO2 | 4.76545 | 333.1442315 | LMFA01090012 | - | 1.260281754 | 0.0171 | - | ↓ |
| (±) -Octanoylcarnitine | C15H29NO4 | 4.76545 | 288.216716 | - | - | 1.364845049 | 0.02295 | - | ↑ |
| Isomucronulatol | C17H18O5 | 4.70595 | 303.1224595 | HMDB0033189;LMPK12080033 | - | 2.931075437 | 6.05E-06 | - | ↑ |
| Dihydroroseoside | C19H32O8 | 4.648616667 | 411.1984139 | HMDB0040614 | - | 2.379730205 | 0.0002532 | - | ↑ |
| Atrolactic acid | C9H10O3 | 4.648616667 | 167.0699019 | - | C05584 | 3.394102327 | 6.98E-09 | map01100;map00350 | ↑ |
| 1, 4-Ipomeadiol | C9H14O3 | 4.5858 | 171.1016314 | HMDB0030471 | - | 1.799198256 | 0.003962 | - | ↑ |
| Small bacteriocin | C18H31NO4 | 4.543966667 | 358.2592693 | HMDB0033503 | - | 2.435781621 | 0.0004144 | - | ↑ |
| 4-formyl Indole | C9H7NO | 4.280816667 | 146.0594334 | - | - | 1.113697121 | 0.007574 | - | ↑ |
| 2- (2-Furanyl) -3-piperidinol | C9H13NO2 | 3.297366667 | 352.2231609 | HMDB0039837 | - | 1.361981416 | 0.0356 | - | ↓ |
| (R) - (+) -2-Pyrrolidone-5-carboxylic acid | C5H7NO3 | 3.07705 | 130.0491236 | - | C02237 | 2.348023083 | 0.002046 | map00471 | ↑ |
| Tyrosyl-Lysine | C15H23N3O4 | 3.07705 | 310.1756636 | HMDB0029110 | - | 2.080221608 | 0.001712 | - | ↑ |
| Oxindole | C8H7NO | 3.008216667 | 134.0589914 | - | C12312 | 3.986310472 | 2.16E-08 | - | ↑ |
| 2-Methylbutyroylcarnitine | C12H23NO4 | 3.008216667 | 246.1695694 | LMFA07070034;HMDB0000378 | - | 1.704978972 | 3.63E-05 | - | ↑ |
| Citreovirenone | C14H16O4 | 2.979216667 | 266.1395095 | HMDB0033515 | - | 1.668334422 | 0.01478 | - | ↑ |
| Tryptophyl-Cysteine | C14H17N3O3S | 2.8824 | 330.0856625 | HMDB0029080 | - | 4.06236885 | 2.45E-12 | - | ↑ |
| 3b, 6a-Dihydroxy-alpha-ionol 9- [apiosyl- (1->6) -glucoside] | C24H40O12 | 2.8524 | 584.2662657 | HMDB0041577 | - | 2.57959506 | 4.60E-05 | - | ↑ |
| Pteroside Z | C21H30O7 | 2.8214 | 436.2366681 | HMDB0032587 | - | 1.330890203 | 0.003993 | - | ↑ |
| Isoleucylproline | C11H20N2O3 | 2.7814 | 211.143161 | HMDB0011174 | - | 2.264505598 | 0.003526 | - | ↓ |
| Pantothenic Acid | C9H17NO5 | 2.7029 | 220.117384 | HMDB0000210 | C00864 | 1.687501085 | 1.28E-06 | map01100;map00770;map00410;map04977 | ↑ |
| O-Adipoylcarnitine | C13H23NO6 | 2.6449 | 290.1596528 | LMFA07070087;HMDB0061677 | - | 3.426928279 | 1.95E-09 | - | ↑ |
| 3, 4, 5-trihydroxy-6- [(3-methylbut-2-enoyl) oxy] oxane-2-carboxylic acid | C11H16O8 | 2.469283333 | 318.1177366 | HMDB0128920 | - | 3.775255732 | 9.78E-09 | - | ↑ |
| N-Acetylproline | C7H11NO3 | 2.336583333 | 199.1070549 | HMDB0094701 | - | 2.955945046 | 5.04E-06 | - | ↑ |
| 1-Methylinosine | C11H14N4O5 | 1.92345 | 283.1035165 | HMDB0002721 | - | 2.7292728 | 8.55E-09 | - | ↑ |
| 7-Methylxanthine | C6H6N4O2 | 1.5768 | 167.0558986 | HMDB0001991 | C16353 | 2.064822126 | 0.001627 | map01100;map00232 | ↑ |
| Threoninyl-Glutamate | C9H16N2O6 | 1.460466667 | 271.0923513 | HMDB0029060 | - | 2.850766201 | 3.86E-10 | - | ↑ |
| N-Methyl-2-pyridone-5-carboxamide (Nudifloramide) | C7H8N2O2 | 1.372983333 | 153.0655681 | - | - | 2.284656698 | 5.07E-08 | - | ↑ |
| Casuarine 6-alpha-D-glucoside | C14H25NO10 | 1.278816667 | 400.1828174 | HMDB0031999 | - | 1.547698567 | 0.01282 | - | ↓ |
| Pyrraline | C12H18N2O4 | 0.866516667 | 219.1128003 | HMDB0033143 | - | 2.390785164 | 0.0048 | - | ↑ |
| S-Adenosylhomocysteine | C14H20N6O5S | 0.76835 | 385.1294099 | HMDB0000939 | C00021 | 2.064695841 | 8.77E-05 | map01100;map00270;map01230 | ↑ |
| Nepsilon-Acetyl-L-lysine | C8H16N2O3 | 0.748516667 | 189.1230747 | - | - | 1.612887074 | 1.30E-07 | - | ↑ |
| (+/-) -Ethyl 2-hydroxy-2-methylbutyrate | C7H14O3 | 0.729183333 | 188.1276023 | HMDB0032269;LMFA07010693 | - | 1.758050269 | 0.0133 | - | ↓ |
| 1-Amino-propan-2-ol | C3H9NO | 0.671016667 | 151.143851 | HMDB0012136 | C05771 | 2.222546124 | 6.53E-05 | - | ↑ |
| N2- (3-Carboxy-2-hydroxy-1-oxopropyl) arginine | C10H18N4O6 | 0.661516667 | 335.0951945 | HMDB0039408 | - | 2.719704343 | 2.68E-06 | - | ↑ |

M/Z, mass-to-charge ratio. Metabolite changes in DKD ESRD group are shown as (↑) for increase or (↓) for decrease.

### Table S2. 192 microbiota -related serum metabolites Between Groups in DKD Patients (p<0.05)

| Metabolites | g_unclassified_f_Ruminococcaceae | g_Faecalicoccus | f_Aerococcaceae | g_Tyzzerella | g_Lachnospiraceae_NC2004_group | g_norank_f_Peptococcaceae | g_Staphylococcus | g_Catenibacillus | g_Olsenella | g_Abiotrophia | g_norank_f_norank_o__Oscillospirales |
| --- | --- | --- | --- | --- | --- | --- | --- | --- | --- | --- | --- |
| Porson | -0.035 | 0.1634 | -0.081 | -0.395* | 0.0944 | 0.117 | 0.1337 | -0.1577 | 0.0764 | -0.0816 | 0.1047 |
| 16-bromo-9E-hexadecenoic acid | -0.1528 | 0.0409 | 0.0427 | -0.461* | -0.1107 | -0.0143 | 0.2589 | -0.2954 | 0.0629 | 0.0427 | 0.0609 |
| Norfentanyl | -0.1966 | 0.1332 | -0.074 | -0.398* | -0.0589 | -0.0877 | 0.1629 | -0.2905 | -0.0078 | -0.0744 | 0.0805 |
| Endomorphin-1 | -0.1708 | 0.2804 | -0.104 | -0.0727 | 0.1218 | -0.247 | 0.4732^*^ | -0.1192 | 0.087 | -0.1041 | -0.0498 |
| Zapoterin | -0.1875 | 0.2859 | -0.174 | -0.432^*^ | 0.0686 | -0.2079 | 0.4504^*^ | -0.1241 | -0.0232 | -0.1748 | -0.2926 |
| Cortisol | -0.1711 | 0.5056^*^ | 0.0058 | -0.1691 | 0.0538 | -0.082 | 0.1917 | -0.2333 | 0.1079 | 0.0058 | -0.1358 |
| 1alpha-hydroxy-25, 26, 27-trinorvitamin D3 24-carboxylic acid | -0.407^*^ | -0.035 | -0.399^*^ | -0.2738 | 0.0242 | -0.2482 | 0.0283 | -0.452^*^ | -0.1108 | -0.399^*^ | -0.0552 |
| 41-O-demethylrapamycin | -0.2795 | 0.0984 | -0.1632 | -0.414^*^ | 0.0254 | -0.263 | -0.1411 | -0.411^*^ | -0.1169 | -0.1632 | -0.224 |
| Soyasaponin III | -0.2613 | 0.1237 | -0.354^*^ | -0.2245 | 0.2608 | -0.381^*^ | 0.3256 | -0.1871 | 0.1144 | -0.354^*^ | -0.2431 |
| Butylparaben | -0.369^*^ | 0.1014 | -0.2946 | -0.151 | 0.2438 | -0.393^*^ | 0.3336 | -0.2792 | 0.0488 | -0.2946 | -0.1266 |
| Isoleucylproline | -0.1872 | 0.1148 | -0.357^*^ | -0.1746 | 0.0893 | -0.398^*^ | 0.1999 | -0.3146 | 0.0673 | -0.357^*^ | -0.2186 |
| 4-ethylamino-6-isopropylamino-1, 3, 5-triazin-2-ol | -0.411^*^ | 0.1178 | -0.385^*^ | -0.401^*^ | 0.1866 | -0.2165 | 0.2252 | -0.317 | -0.054 | -0.385^*^ | -0.3047 |
| Carboprost Tromethamine | -0.2739 | 0.0895 | -0.2148 | -0.416^*^ | 0.1225 | -0.2527 | 0.1709 | -0.1257 | -0.1483 | -0.2148 | -0.1702 |
| L-Oxalylalbizziine | -0.2949 | 0.0702 | -0.1917 | -0.458^*^ | -0.0708 | -0.293 | 0.3676^*^ | -0.2677 | -0.0614 | -0.1917 | -0.1934 |
| 2- (2-Furanyl) -3-piperidinol | -0.3348 | 0.0027 | -0.1437 | -0.374^*^ | -0.0229 | -0.359^*^ | 0.3427^*^ | -0.3162 | 0.0394 | -0.1437 | -0.0639 |
| Glycerophosphocholine | -0.2882 | 0.0712 | -0.165 | -0.379^*^ | 0.2695 | -0.0817 | 0.2607 | -0.3275 | 0.1448 | -0.165 | -0.3182 |
| 28-Glucosyl-19 (29) -dehydroursolic acid 3-arabinoside | -0.429^*^ | 0.1297 | -0.1367 | -0.386^*^ | 0.1482 | -0.448^*^ | 0.249 | -0.362^*^ | 0.1172 | -0.1367 | -0.3133 |
| Solacauline | -0.355^*^ | 0.0771 | -0.2478 | -0.2856 | 0.2278 | -0.485^*^ | 0.222 | -0.3173 | 0.143 | -0.2478 | -0.381^*^ |
| PS (DiMe (9, 3) /MonoMe (13, 5) ) | -0.406^*^ | 0.1366 | -0.2475 | -0.438^*^ | 0.2123 | -0.396^*^ | 0.2197 | -0.3004 | 0.047 | -0.2475 | -0.439^*^ |
| Melanostatin | -0.3194 | -0.028 | -0.0816 | -0.463^*^ | 0.1115 | -0.1918 | 0.1967 | -0.522^*^ | 0.2923 | -0.0816 | -0.0804 |
| 4-Hydroxy-5-phenyltetrahydro-1, 3-oxazin-2-one | -0.2685 | 0.3067 | -0.0146 | -0.456^*^ | 0.0725 | -0.333 | 0.0627 | -0.432^*^ | 0.2302 | -0.0146 | -0.0453 |
| Hovenine A | -0.2915 | 0.0761 | -0.1182 | -0.464^*^ | 0.2273 | -0.3263 | 0.0285 | -0.372^*^ | 0.2054 | -0.1182 | -0.041 |
| Epsilon-caprolactam | -0.2234 | 0.2457 | -0.1437 | -0.3331 | 0.1793 | -0.3295 | 0.0816 | -0.394^*^ | 0.2629 | -0.1437 | -0.1029 |
| NIPECOTIC ACID | -0.2912 | 0.2353 | -0.2183 | -0.409^*^ | 0.1822 | -0.329 | 0.1074 | -0.3278 | 0.2654 | -0.2183 | -0.1335 |
| Brassica napus non-fluorescent chlorophyll catabolite 3 | -0.293 | 0.2735 | 0.042 | -0.458^*^ | 0.1474 | -0.3212 | 0.2897 | -0.353^*^ | 0.0399 | 0.042 | -0.1781 |
| 4-Hydroxystachydrine | -0.393^*^ | 0.1317 | -0.035 | -0.441^*^ | 0.2095 | -0.602^*^ | 0.0456 | -0.398^*^ | 0.2042 | -0.035 | -0.2491 |
| 1-Piperidine carboxylic acid | -0.398^*^ | 0.2051 | -0.1393 | -0.500^*^ | 0.1869 | -0.404^*^ | 0.1235 | -0.481^*^ | 0.2566 | -0.1393 | -0.248 |
| 2-Amino-4-ethoxy-3-hydroxybutanoic acid | 0.1656 | 0.3112 | -0.0943 | -0.035 | 0.2204 | -0.2133 | -0.0613 | -0.2298 | 0.5048^*^ | -0.0943 | 0.0588 |
| O-Phosphotyrosine | -0.0314 | 0.1763 | -0.1013 | -0.1037 | 0.2601 | -0.2822 | -0.0499 | -0.2177 | 0.4787^*^ | -0.1013 | 0.0457 |
| Glucosamine-1P | -0.2243 | 0.2859 | -0.0723 | -0.2282 | 0.1376 | -0.3196 | -0.065 | -0.3041 | 0.3429^*^ | -0.0723 | -0.073 |
| 1- (beta-D-Ribofuranosyl) -1, 4-dihydronicotinamide | -0.0872 | 0.2829 | -0.124 | -0.2659 | 0.1987 | -0.2813 | 0.0097 | -0.2571 | 0.4752^*^ | -0.124 | -0.0876 |
| L-Tryptophan | -0.0351 | 0.2259 | -0.0621 | -0.348^*^ | 0.0786 | -0.1682 | 0.1508 | -0.1931 | 0.1888 | -0.0621 | -0.1844 |
| Neuromedin B (1-3) | -0.1321 | 0.1431 | 0.0042 | -0.2488 | 0.159 | -0.1253 | 0.0293 | -0.362^*^ | 0.3391^*^ | 0.0042 | -0.1309 |
| Tyrosol 4-sulfate | 0.7946^*^ | 0.1887 | 0.0645 | 0.2128 | -0.2118 | 0.5878^*^ | 0.0424 | 0.4515^*^ | 0.1425 | 0.0645 | 0.4331^*^ |
| 1, 4-Ipomeadiol | 0.542^*^ | 0.0027 | 0.0171 | 0.4909^*^ | -0.1481 | 0.4211^*^ | 0.0392 | 0.5423^*^ | 0.1554 | 0.0171 | 0.4249^*^ |
| P-Tolyl Sulfate | 0.6809^*^ | 0.0741 | 0.0964 | 0.6563^*^ | -0.2628 | 0.5495^*^ | -0.1131 | 0.4263^*^ | 0.0281 | 0.0964 | 0.4707^*^ |
| P-cresol | 0.6672^*^ | 0.0741 | 0.1032 | 0.6391^*^ | -0.2623 | 0.5438^*^ | -0.1374 | 0.4052^*^ | 0.0347 | 0.1032 | 0.4672^*^ |
| {3- [2- (3-hydroxy-5-methoxyphenyl) ethyl] phenyl} oxidanesulfonic acid | 0.3586^*^ | -0.0087 | -0.1613 | 0.3427^*^ | -0.0476 | 0.4113^*^ | -0.243 | 0.4797^*^ | -0.1186 | -0.1613 | 0.0915 |
| 5'-Carboxy-gamma-chromanol | 0.2859 | -0.1024 | -0.0524 | 0.2212 | -0.1408 | 0.3032 | -0.1927 | 0.37^*^ | -0.1142 | -0.0524 | 0.1376 |
| (R) - (+) -2-Pyrrolidone-5-carboxylic acid | 0.2316 | -0.092 | 0.0345 | 0.4027^*^ | -0.2431 | 0.4401^*^ | -0.0501 | 0.2005 | -0.1739 | 0.0345 | 0.3535^*^ |
| 2- (2-Phenylacetoxy)  propionylglycine | 0.2588 | 0.0092 | 0.0688 | 0.3614^*^ | -0.2709 | 0.4457^*^ | -0.058 | 0.0729 | -0.1674 | 0.0688 | 0.2828 |
| Tyrosyl-Lysine | 0.3564^*^ | -0.0379 | 0.0441 | 0.4063^*^ | -0.3244 | 0.5275^*^ | -0.0099 | 0.2471 | -0.1963 | 0.0441 | 0.4053^*^ |
| N- [(3a, 5b, 7b) -7-hydroxy-24-oxo-3- (sulfooxy) cholan-24-yl] -Glycine | 0.3737^*^ | -0.0548 | 0.0445 | 0.4392^*^ | -0.2002 | 0.4733^*^ | 0.1783 | 0.2897 | -0.213 | 0.0445 | 0.4375^*^ |
| Glutamylisoleucine | 0.4108^*^ | 0.0771 | 0.067 | 0.213 | -0.1501 | 0.3645^*^ | 0.2547 | 0.2773 | 0.0015 | 0.067 | 0.3565^*^ |
| Pyrocatechol sulfate | 0.4132^*^ | -0.1505 | -0.0477 | 0.2983 | -0.1728 | 0.3815^*^ | 0.0747 | 0.3372 | -0.1257 | -0.0477 | 0.3684^*^ |
| (3b, 4b, 11b, 14b) -11-Ethoxy-3, 4-epoxy-14-hydroxy-12-cyathen-15-al 14-xyloside | 0.2616 | -0.1356 | 0.0276 | 0.1474 | -0.1211 | 0.3982^*^ | -0.036 | 0.2601 | -0.0684 | 0.0276 | 0.3674^*^ |
| 5-Heptyltetrahydro-2-oxo-3-furancarboxylic acid | 0.2733 | -0.432^*^ | 0.1798 | 0.3062 | -0.2063 | 0.3785^*^ | -0.091 | 0.3489^*^ | -0.2373 | 0.1798 | 0.2125 |
| Dodecanedioic acid | 0.2918 | -0.537^*^ | 0.0816 | 0.2603 | -0.1674 | 0.3921^*^ | -0.0295 | 0.3106 | -0.3228 | 0.0816 | 0.2235 |
| 1-Amino-propan-2-ol | 0.3081 | -0.2799 | 0.0691 | 0.3741^*^ | -0.0434 | 0.4674^*^ | 0.001 | 0.2994 | -0.2901 | 0.0691 | 0.3535^*^ |
| Ustiloxin D | 0.2215 | -0.2993 | 0.0973 | 0.3074 | 0.0466 | 0.3611^*^ | -0.088 | 0.3484^*^ | -0.2491 | 0.0973 | 0.1895 |
| 1, 3-Dihydro- (2H) -indol-2-one | 0.4004^*^ | -0.1783 | 0.143 | 0.1619 | -0.1871 | 0.6464^*^ | -0.0823 | 0.3855^*^ | -0.1275 | 0.143 | 0.2565 |
| N-Acetylarylamine | 0.4276^*^ | -0.2671 | 0.181 | 0.2577 | -0.1851 | 0.5836^*^ | -0.0933 | 0.4103^*^ | -0.1108 | 0.181 | 0.3818^*^ |
| Citreovirenone | 0.3463^*^ | -0.2304 | -0.0702 | 0.2993 | -0.2211 | 0.5428^*^ | -0.221 | 0.4191^*^ | -0.2086 | -0.0702 | 0.2826 |
| 3, 4, 5-trihydroxy-6- [(3-methylbut-2-enoyl) oxy] oxane-2-carboxylic acid | 0.3064 | -0.2378 | 0.0236 | 0.4436^*^ | -0.1624 | 0.5233^*^ | -0.2587 | 0.3225 | -0.2403 | 0.0236 | 0.1848 |
| 1alpha, 18-dihydroxyvitamin D3 | 0.3861^*^ | 0.088 | 0.1488 | 0.4208^*^ | -0.1654 | 0.3576^*^ | -0.2368 | 0.4125^*^ | -0.0051 | 0.1488 | 0.2218 |
| 4-Hydroxy-5- (phenyl) -valeric acid-O-glucuronide | 0.4643^*^ | 0.0275 | 0.0113 | 0.4352^*^ | -0.2532 | 0.4398^*^ | -0.2738 | 0.2894 | -0.0183 | 0.0113 | 0.1547 |
| O-methoxycatechol-O-sulphate | 0.5217^*^ | -0.0771 | 0.1156 | 0.4957^*^ | -0.3064 | 0.3275 | -0.2225 | 0.3215 | -0.1214 | 0.1156 | 0.2243 |
| 4-Methylcatechol | 0.5054^*^ | -0.1426 | 0.1008 | 0.5074^*^ | -0.2514 | 0.3515^*^ | -0.2391 | 0.3665^*^ | -0.1807 | 0.1008 | 0.2093 |
| 9-Oxo-nonanoic acid | 0.4447^*^ | -0.2601 | 0.1516 | 0.4097^*^ | -0.3082 | 0.4879^*^ | -0.2388 | 0.3177 | -0.1149 | 0.1516 | 0.3382 |
| Phaseolic acid | 0.3835^*^ | -0.1798 | 0.1944 | 0.4143^*^ | -0.2482 | 0.5455^*^ | -0.2259 | 0.3731^*^ | -0.1818 | 0.1944 | 0.3094 |
| 17-alpha, 21-dihydroxy-11, 20-dioxo-5-beta-pregnan-3-alpha-yl-beta-d-glucuronide | 0.4147^*^ | -0.209 | 0.2244 | 0.3794^*^ | -0.3069 | 0.329 | -0.1753 | 0.3718^*^ | -0.0938 | 0.2244 | 0.3504^*^ |
| N-di-Demethyl roxithromycin | 0.3384 | -0.1371 | 0.1759 | 0.4527^*^ | -0.1889 | 0.4086^*^ | -0.1689 | 0.3175 | -0.0968 | 0.1759 | 0.3426^*^ |
| Celecoxib glucuronide | 0.2411 | -0.1664 | 0.1694 | 0.5738^*^ | -0.1454 | 0.1507 | -0.2366 | 0.3435^*^ | -0.0488 | 0.1694 | 0.1795 |
| Oxindole | 0.2322 | -0.2869 | 0.2846 | 0.4815^*^ | -0.1043 | 0.2953 | -0.1952 | 0.3387 | -0.2031 | 0.2846 | 0.2825 |
| 5a-Androst-3-en-17-one | 0.2346 | -0.3375 | 0.2739 | 0.4935^*^ | -0.1265 | 0.2551 | -0.1518 | 0.281 | -0.187 | 0.2739 | 0.339^*^ |
| 5beta-1, 3, 7 (11) -Eudesmatrien-8-one | 0.2736 | -0.377^*^ | 0.2682 | 0.323 | -0.0806 | 0.4362^*^ | -0.3051 | 0.3384 | -0.2458 | 0.2682 | 0.1176 |
| Osmundalactone | 0.2226 | -0.1485 | 0.1476 | 0.3533^*^ | -0.1661 | 0.343^*^ | -0.3361 | 0.375^*^ | -0.128 | 0.1476 | 0.2305 |
| Ribothymidine | 0.183 | -0.2973 | 0.197 | 0.4498^*^ | -0.2189 | 0.2355 | -0.2993 | 0.201 | -0.1931 | 0.197 | 0.2581 |
| 3, 4, 5-trihydroxy-6- [3- (4-methoxyphenyl) propoxy] oxane-2-carboxylic acid | 0.1485 | -0.2725 | 0.1975 | 0.4022^*^ | -0.1635 | 0.2594 | -0.466^*^ | 0.2075 | -0.1132 | 0.1975 | 0.1794 |
| Cucurbic acid | 0.2907 | -0.1981 | 0.2149 | 0.1941 | -0.400^*^ | 0.5214^*^ | -0.1632 | 0.0328 | -0.1111 | 0.2149 | 0.2586 |
| 3-hydroxy-3- (3-hydroxyphenyl) propanoic acid-O-sulphate | 0.1865 | -0.1217 | 0.1641 | 0.1561 | -0.2317 | 0.4704^*^ | -0.2361 | 0.2209 | -0.2041 | 0.1641 | 0.3803^*^ |
| S-Adenosylhomocysteine | 0.2639 | -0.2051 | 0.1421 | 0.1888 | -0.2233 | 0.6236^*^ | -0.2738 | 0.2477 | -0.1878 | 0.1421 | 0.1755 |
| Trans-Dehydroandrosterone | 0.432^*^ | -0.3072 | 0.2376 | 0.4438^*^ | -0.3314 | 0.4319^*^ | -0.0216 | 0.3497^*^ | -0.1803 | 0.2376 | 0.436^*^ |
| 3-Indole carboxylic acid glucuronide | 0.4068^*^ | -0.3092 | 0.2179 | 0.471^*^ | -0.3223 | 0.4484^*^ | -0.1431 | 0.4258^*^ | -0.2044 | 0.2179 | 0.4006^*^ |
| Furocoumarinic acid glucoside | 0.3577^*^ | -0.3052 | 0.3198 | 0.4004^*^ | -0.366^*^ | 0.3844^*^ | 0.0027 | 0.2635 | -0.1465 | 0.3198 | 0.3328 |
| 5-Androstene-3b, 16b, 17a-triol | 0.3177 | -0.3176 | 0.3187 | 0.441^*^ | -0.2404 | 0.345^*^ | -0.0637 | 0.3663^*^ | -0.1765 | 0.3187 | 0.3565^*^ |
| N-METHYLANTHRANILIC ACID | 0.3997^*^ | -0.362^*^ | 0.216 | 0.3645^*^ | -0.3277 | 0.3507^*^ | -0.2716 | 0.2828 | -0.0277 | 0.216 | 0.3855^*^ |
| 5alpha-Androst-16-en-3alpha-ol | 0.2871 | -0.2537 | 0.169 | 0.4419^*^ | -0.2574 | 0.2887 | -0.152 | 0.1645 | -0.1524 | 0.169 | 0.4612^*^ |
| Indoxylsulfuric acid | 0.2733 | -0.2715 | 0.2529 | 0.4023^*^ | -0.2926 | 0.3052 | -0.2113 | 0.249 | -0.2496 | 0.2529 | 0.3988^*^ |
| Dihydrojasmonic acid | 0.3194 | -0.2452 | 0.3405^*^ | 0.1035 | -0.1975 | 0.465^*^ | 0.1453 | 0.3227 | -0.0443 | 0.3405^*^ | 0.2673 |
| (Z) -3-Oxo-2- (2-pentenyl) -1-cyclopenteneacetic acid | 0.321 | -0.0756 | 0.331 | 0.1206 | -0.2695 | 0.3125 | 0.0704 | 0.4009^*^ | -0.0505 | 0.331 | 0.2762 |
| 3, 4-Methylenesebacic acid | 0.3375 | -0.344^*^ | 0.296 | 0.2469 | -0.2073 | 0.2918 | 0.0444 | 0.2149 | -0.1313 | 0.296 | 0.4308^*^ |
| Jasmolone | 0.3184 | -0.2368 | 0.327 | 0.2366 | -0.2177 | 0.4174^*^ | 0.0898 | 0.2718 | -0.0457 | 0.327 | 0.4684^*^ |
| Marmelolactone A | 0.3219 | -0.3181 | 0.401^*^ | 0.2796 | -0.2471 | 0.381^*^ | 0.0935 | 0.2755 | -0.1448 | 0.401^*^ | 0.3676^*^ |
| Benzenebutanoic acid | 0.4043^*^ | -0.1783 | 0.3227 | 0.3314 | -0.368^*^ | 0.4182^*^ | 0.0174 | 0.3563^*^ | -0.1113 | 0.3227 | 0.501^*^ |
| 3, 4-Methylene suberic acid | 0.3746^*^ | -0.2199 | 0.4172^*^ | 0.3573^*^ | -0.2532 | 0.3989^*^ | -0.0871 | 0.3363 | -0.0865 | 0.4172^*^ | 0.4097^*^ |
| 6- (4-ethyl-5-hydroxy-2-methoxyphenoxy) -3, 4, 5-trihydroxyoxane-2-carboxylic acid | 0.3689^*^ | -0.2304 | 0.3057 | 0.392^*^ | -0.2359 | 0.3874^*^ | -0.1151 | 0.3206 | -0.1036 | 0.3057 | 0.4434^*^ |
| Muramic acid | 0.3849^*^ | -0.2611 | 0.3268 | 0.2628 | -0.2924 | 0.3968^*^ | -0.094 | 0.3623^*^ | -0.1348 | 0.3268 | 0.4171^*^ |
| N2- (3-Carboxy-2-hydroxy-1-oxopropyl) arginine | 0.3014 | -0.2646 | 0.4239^*^ | 0.2001 | -0.3599^*^ | 0.5002^*^ | -0.1391 | 0.4387^*^ | -0.3457^*^ | 0.4239^*^ | 0.191 |
| Gamma-Glutamylproline | 0.3588^*^ | -0.382^*^ | 0.3572^*^ | 0.2613 | -0.3368 | 0.4677^*^ | -0.1664 | 0.3136 | -0.217 | 0.3572^*^ | 0.1508 |
| N-Acetyl-L-alanine | 0.2228 | -0.1768 | 0.3268 | 0.1074 | -0.3212 | 0.4189^*^ | -0.2423 | 0.2939 | -0.1993 | 0.3268 | 0.108 |
| Ascorbic acid-2-sulfate | 0.2876 | -0.1937 | 0.3803^*^ | 0.2024 | -0.2662 | 0.3211 | -0.1257 | 0.3236 | -0.3039 | 0.3803^*^ | 0.2921 |
| Ganoderic acid H | 0.3168 | -0.1991 | 0.4008^*^ | 0.2479 | -0.347^*^ | 0.4534^*^ | -0.181 | 0.3009 | -0.173 | 0.4008^*^ | 0.273 |
| 3b, 6a-Dihydroxy-alpha-ionol 9- [apiosyl- (1->6) -glucoside] | 0.3442 | -0.2923 | 0.1827 | 0.1947 | -0.419^*^ | 0.4061^*^ | -0.0248 | 0.3198 | -0.2112 | 0.1827 | 0.0358 |
| Tetrahydrogestrinone | 0.1758 | -0.2869 | 0.254 | 0.0413 | -0.2473 | 0.4894^*^ | -0.1002 | 0.1794 | -0.1971 | 0.254 | 0.2372 |
| Creatine riboside | 0.2902 | -0.366^*^ | 0.2825 | 0.2224 | -0.1366 | 0.5371^*^ | -0.1094 | 0.2175 | -0.1773 | 0.2825 | 0.1908 |
| Dihydroroseoside | 0.2408 | -0.1029 | 0.3366 | 0.2267 | -0.2858 | 0.1865 | -0.218 | 0.1965 | -0.1506 | 0.3366 | 0.3738^*^ |
| Artemidiol | 0.2835 | -0.0404 | 0.3708^*^ | 0.4248^*^ | -0.3144 | 0.1534 | -0.1327 | 0.2792 | -0.2903 | 0.3708^*^ | 0.4234^*^ |
| Threoninyl-Glutamate | 0.4499^*^ | -0.1808 | 0.3479^*^ | 0.3888^*^ | -0.3102 | 0.3923^*^ | -0.1902 | 0.2781 | -0.091 | 0.3479^*^ | 0.2419 |
| N-Acetylproline | 0.4603^*^ | -0.1897 | 0.3358 | 0.3824^*^ | -0.457^*^ | 0.4029^*^ | -0.1295 | 0.3513^*^ | -0.1548 | 0.3358 | 0.1771 |
| Atrolactic acid | 0.4312^*^ | -0.2264 | 0.36^*^ | 0.4339^*^ | -0.2645 | 0.329 | -0.2396 | 0.412^*^ | -0.1333 | 0.36^*^ | 0.3257 |
| 1-Methylinosine | 0.3675^*^ | -0.3127 | 0.3449^*^ | 0.3956^*^ | -0.367^*^ | 0.4051^*^ | -0.1825 | 0.3802^*^ | -0.2396 | 0.3449^*^ | 0.3186 |
| Tryptophyl-Cysteine | 0.3157 | -0.2561 | 0.3449^*^ | 0.3609^*^ | -0.2754 | 0.3805^*^ | -0.2495 | 0.3369 | -0.1946 | 0.3449^*^ | 0.3094 |
| 7-Aminomethyl-7-carbaguanine | 0.2977 | -0.2849 | 0.3597^*^ | 0.3501^*^ | -0.3341 | 0.3357 | -0.1605 | 0.3272 | -0.2199 | 0.3597^*^ | 0.263 |
| Blepharin | 0.3922^*^ | -0.1198 | 0.5387^*^ | 0.3991^*^ | -0.457^*^ | 0.236 | -0.218 | 0.2959 | -0.0611 | 0.5387^*^ | 0.3651^*^ |
| Furaneol 4- (6-malonylglucoside) | 0.2958 | -0.2284 | 0.5419^*^ | 0.3955^*^ | -0.341^*^ | 0.2529 | -0.0387 | 0.3361 | -0.1813 | 0.5419^*^ | 0.2557 |
| 8-Hydroxyoctanoate | 0.2876 | -0.0965 | 0.4274^*^ | 0.3989^*^ | -0.254 | 0.2386 | -0.0471 | 0.2778 | -0.1421 | 0.4274^*^ | 0.3078 |
| [4- (4-hydroxy-3-methoxyphenyl) -2-oxobutoxy] sulfonic acid | 0.3788^*^ | -0.1059 | 0.251 | 0.4422^*^ | -0.1767 | 0.2743 | -0.3234 | 0.2411 | -0.0025 | 0.251 | 0.4296^*^ |
| (2E, 4E) -2, 7-Dimethyl-2, 4-octadienedioic acid | 0.4247^*^ | -0.0528 | 0.3461^*^ | 0.4517^*^ | -0.1955 | 0.4517^*^ | -0.158 | 0.4209^*^ | -0.0251 | 0.3461^*^ | 0.385^*^ |
| Indolylacryloylglycine | 0.4326^*^ | -0.0315 | 0.3148 | 0.4308^*^ | -0.157 | 0.4664^*^ | -0.2683 | 0.3243 | -0.0051 | 0.3148 | 0.41^*^ |
| 3- (6, 7-dimethoxy-2H-1, 3-benzodioxol-5-yl) prop-2-en-1-ol | 0.3532^*^ | -0.1257 | 0.4124^*^ | 0.1916 | -0.1169 | 0.4935^*^ | -0.1572 | 0.2479 | 0.0628 | 0.4124^*^ | 0.3091 |
| Tetrahydrocortisone | 0.328 | -0.0761 | 0.2211 | 0.1677 | -0.2614 | 0.4359^*^ | -0.1761 | 0.2601 | -0.0503 | 0.2211 | 0.2803 |
| Dinorpromazine | 0.4063^*^ | -0.1684 | 0.2965 | 0.2522 | -0.2212 | 0.3662^*^ | -0.2272 | 0.2448 | -0.0676 | 0.2965 | 0.1826 |
| 1-Hydroxy-2, 12, 15-heneicosatrien-4-one | 0.3378 | -0.1763 | 0.2825 | 0.3086 | -0.318 | 0.3712^*^ | -0.2016 | 0.2015 | -0.0968 | 0.2825 | 0.2369 |
| Cytokinin B | 0.5759^*^ | -0.0042 | 0.2619 | 0.2952 | -0.380^*^ | 0.394^*^ | 0.0774 | 0.168 | 0.1709 | 0.2619 | 0.3754^*^ |
| Dihyroxy-1H-indole glucuronide I | 0.3906^*^ | -0.1867 | 0.2663 | 0.3946^*^ | -0.434^*^ | 0.4605^*^ | -0.0293 | 0.1286 | -0.0515 | 0.2663 | 0.2856 |
| 4-Ethyl-2-hydroxy-3-methyl-2-cyclopenten-1-one | 0.4499^*^ | -0.1287 | 0.3361 | 0.3968^*^ | -0.3126 | 0.543^*^ | -0.0246 | 0.2089 | 0.0083 | 0.3361 | 0.3439^*^ |
| Hippuric acid | 0.512^*^ | -0.1758 | 0.3628^*^ | 0.2723 | -0.3065 | 0.5778^*^ | -0.0521 | 0.4983^*^ | -0.1162 | 0.3628^*^ | 0.4806^*^ |
| 2, 3-Methylene suberic acid | 0.4486^*^ | -0.1287 | 0.4042^*^ | 0.3388 | -0.3176 | 0.4704^*^ | -0.1801 | 0.4434^*^ | -0.0144 | 0.4042^*^ | 0.3958^*^ |
| Pantothenic Acid | 0.5215^*^ | -0.0712 | 0.3762^*^ | 0.3814^*^ | -0.2431 | 0.5346^*^ | -0.2004 | 0.4505^*^ | -0.0495 | 0.3762^*^ | 0.3306 |
| BENZYLBUTYLPHTHALATE | 0.4892^*^ | -0.1034 | 0.2642 | 0.3617^*^ | -0.2209 | 0.4564^*^ | 0.0441 | 0.4889^*^ | 0.0784 | 0.2642 | 0.361^*^ |
| 2-Hydroxyacetaminophen sulfate | 0.4607^*^ | -0.1956 | 0.2118 | 0.4366^*^ | -0.266 | 0.5^*^ | -0.0129 | 0.2395 | -0.0223 | 0.2118 | 0.4439^*^ |
| 6-dehydrotestosterone | 0.4623^*^ | -0.2056 | 0.1796 | 0.4464^*^ | -0.2614 | 0.4701^*^ | 0.0216 | 0.3834^*^ | -0.1343 | 0.1796 | 0.4952^*^ |
| 5-Sulfoxymethylfurfural | -0.0916 | -0.276 | -0.2462 | 0.0668 | 0.0298 | 0.1104 | -0.385^*^ | 0.1018 | 0.0272 | -0.2462 | -0.0792 |
| DG (18:1 (11Z) /20:4 (8Z, 11Z, 14Z, 17Z) /0:0) | 0.0532 | -0.216 | 0.0445 | 0.158 | -0.0003 | 0.0618 | -0.392^*^ | 0.0178 | 0.1483 | 0.0445 | -0.1797 |
| 7-Hydroxy-1, 7-bis (4-hydroxy-3-methoxyphenyl) -1-heptene-3, 5-dione | 0.1853 | -0.2998 | 0.0767 | 0.2534 | -0.0554 | 0.378^*^ | -0.2279 | 0.1128 | -0.1154 | 0.0767 | 0.298 |
| Dihydro-3-coumaric acid | 0.1191 | -0.1133 | 0.0988 | 0.4996^*^ | 0.1215 | 0.1594 | -0.2178 | 0.2031 | 0.0736 | 0.0988 | 0.2918 |
| 2- [2, 4, 6-trihydroxy-3- (3-methylbut-2-en-1-yl) phenyl] acetic acid | 0.0424 | -0.369^*^ | 0.1129 | 0.141 | -0.229 | 0.0731 | -0.2076 | 0.0601 | -0.177 | 0.1129 | 0.1643 |
| Trans-3-Hydroxycinnamate | -0.0891 | 0.0057 | 0.5217^*^ | -0.1871 | -0.1602 | 0.0167 | -0.1094 | 0.0646 | 0.0272 | 0.5217^*^ | 0.1338 |
| 15beta-Hydroxydesogestrel | 0.0247 | 0.1277 | 0.1467 | 0.0596 | -0.444^*^ | -0.2246 | 0.3589^*^ | 0.0187 | -0.2502 | 0.1467 | 0.0867 |
| 3-hydroxydodecanoyl carnitine | 0.2406 | 0.0667 | 0.3097 | 0.163 | -0.382^*^ | 0.0872 | -0.0097 | -0.021 | 0.1516 | 0.3097 | 0.3488^*^ |
| Cinncassiol D1 glucoside | 0.1334 | -0.1694 | 0.2851 | 0.2568 | -0.158 | 0.0609 | 0.187 | 0.0267 | 0.0314 | 0.2851 | 0.355^*^ |
| [2-methoxy-4- (prop-2-en-1-yl) phenyl] oxidanesulfonic acid | 0.0857 | -0.1337 | 0.1277 | 0.2102 | -0.2826 | 0.163 | -0.411^*^ | 0.1002 | -0.2165 | 0.1277 | 0.1333 |
| L- (-) -3-Phenyllactic acid | -0.0379 | -0.2145 | 0.1386 | -0.0151 | -0.381^*^ | -0.0071 | -0.187 | -0.1689 | -0.1991 | 0.1386 | -0.0634 |
| Armexifolin | 0.2445 | 0.1322 | 0.279 | 0.0202 | -0.500^*^ | 0.1729 | -0.123 | 0.0737 | -0.1322 | 0.279 | 0.0631 |
| 6-Methylmercaptopurine | 0.1222 | -0.0508 | 0.3036 | -0.0074 | -0.441^*^ | 0.1003 | -0.1711 | -0.1097 | -0.1657 | 0.3036 | -0.0162 |
| Alfalone | 0.1701 | -0.1163 | 0.273 | 0.1121 | -0.496^*^ | 0.1591 | -0.218 | 0.0231 | -0.1759 | 0.273 | 0.0503 |
| Prolyl-Alanine | 0.1954 | -0.2353 | 0.2795 | 0.2754 | -0.498^*^ | 0.146 | 0.0236 | 0.1692 | -0.2272 | 0.2795 | -0.01 |
| (2-Methoxyethoxy) propanoic acid | 0.1435 | -0.2685 | 0.3333 | 0.164 | -0.383^*^ | 0.3095 | -0.127 | 0.0234 | -0.186 | 0.3333 | -0.0039 |
| N-Methyl-2-pyridone-5-carboxamide (Nudifloramide) | 0.2924 | -0.1872 | 0.4365^*^ | 0.1976 | -0.428^*^ | 0.2064 | -0.2406 | 0.2442 | -0.2125 | 0.4365^*^ | 0.1064 |
| N-Acetyl gemifloxacin | 0.1926 | -0.1337 | 0.2089 | 0.2788 | -0.566^*^ | 0.2812 | -0.3269 | 0.1903 | -0.1848 | 0.2089 | 0.0593 |
| Nepsilon-Acetyl-L-lysine | 0.0289 | -0.384^*^ | 0.4102^*^ | 0.2298 | -0.499^*^ | 0.0886 | -0.2257 | 0.0645 | -0.364^*^ | 0.4102^*^ | 0.0941 |
| Isoquinoline | 0.0543 | -0.3355 | 0.3723^*^ | 0.225 | -0.428^*^ | 0.1145 | -0.1672 | 0.1663 | -0.422^*^ | 0.3723^*^ | 0.1624 |
| Indole-3 acetic acid | 0.1825 | -0.357^*^ | 0.2258 | 0.1724 | -0.561^*^ | 0.1244 | -0.1863 | 0.1975 | -0.398^*^ | 0.2258 | 0.1666 |
| 2-Methylbutyroylcarnitine | 0.2254 | -0.1287 | 0.2568 | 0.2503 | -0.425^*^ | 0.1419 | -0.2559 | 0.2125 | -0.3119 | 0.2568 | 0.1524 |
| Dopamine glucuronide | 0.1401 | -0.2175 | 0.2621 | 0.2894 | -0.349^*^ | 0.2347 | -0.2614 | 0.2409 | -0.352^*^ | 0.2621 | 0.2187 |
| 4-formyl Indole | 0.1139 | -0.2418 | -0.0014 | 0.1167 | -0.389^*^ | -0.1106 | 0.0077 | 0.1049 | -0.3132 | -0.0014 | 0.2357 |
| Gamma-CEHC | 0.1705 | -0.2189 | 0.0355 | 0.1535 | -0.3304 | 0.2093 | 0.1332 | 0.1147 | -0.348^*^ | 0.0355 | 0.4213^*^ |
| 2, 6-Dimethyl-4-hydroxybenzaldehyde | 0.1746 | -0.1728 | 0.0554 | 0.1864 | -0.363^*^ | 0.1901 | 0.122 | 0.1415 | -0.366^*^ | 0.0554 | 0.4138^*^ |
| Gibberellin A4 glucosyl ester | 0.0889 | -0.2085 | 0.0359 | 0.2284 | -0.2243 | 0.1229 | 0.0164 | 0.3392^*^ | -0.1792 | 0.0359 | 0.1452 |
| Pteroside Z | 0.1879 | -0.2938 | 0.0412 | 0.1724 | -0.0606 | 0.2776 | -0.0315 | 0.4544^*^ | -0.1333 | 0.0412 | 0.23 |
| Portulacaxanthin II | 0.2461 | -0.2026 | 0.1214 | 0.4467^*^ | -0.388^*^ | 0.3177 | -0.1679 | 0.3196 | -0.1773 | 0.1214 | 0.0557 |
| 1-Methyluric acid | 0.1901 | -0.2214 | 0.0661 | 0.4318^*^ | -0.1994 | 0.2455 | -0.1649 | 0.2752 | -0.1735 | 0.0661 | 0.0943 |
| Allixin | 0.1635 | -0.2125 | 0.2005 | 0.2589 | -0.384^*^ | 0.1497 | -0.0893 | 0.1091 | -0.2119 | 0.2005 | 0.4065^*^ |
| Diosbulbin H | 0.2649 | -0.2512 | 0.1324 | 0.1993 | -0.363^*^ | 0.3199 | -0.0863 | 0.2291 | -0.1609 | 0.1324 | 0.4058^*^ |
| (3b, 16b, 20R) -Pregn-5-ene-3, 16, 20-triol 3-glucoside | 0.2137 | -0.1996 | 0.1687 | 0.2652 | -0.194 | 0.3495^*^ | -0.093 | 0.1855 | -0.169 | 0.1687 | 0.2998 |
| Nepetaside | 0.2789 | -0.1857 | 0.1645 | 0.3038 | -0.2399 | 0.2776 | 0.0231 | 0.2071 | -0.1139 | 0.1645 | 0.3413^*^ |
| Verbasoside | 0.2308 | -0.2289 | 0.0498 | 0.4004^*^ | -0.2614 | 0.3202 | -0.1706 | 0.3068 | -0.1629 | 0.0498 | 0.3506^*^ |
| 14-Dihydroxycornestin | 0.2602 | -0.3023 | -0.0062 | 0.2819 | -0.2766 | 0.219 | -0.068 | 0.2328 | -0.2893 | -0.0062 | 0.3478^*^ |
| N-Acetylcystathionine | 0.1579 | -0.531^*^ | 0.1034 | 0.268 | -0.339^*^ | 0.2583 | -0.0848 | 0.0262 | -0.2662 | 0.1034 | 0.1962 |
| Isomucronulatol | 0.1447 | -0.394^*^ | 0.2155 | 0.3768^*^ | -0.2256 | 0.186 | -0.1952 | 0.2981 | -0.400^*^ | 0.2155 | 0.2354 |
| Indoleacetyl glutamine | 0.0631 | -0.354^*^ | 0.1437 | 0.2075 | -0.2759 | 0.364^*^ | -0.2289 | 0.2671 | -0.375^*^ | 0.1437 | 0.1408 |
| 2-Cinnamoyl-1-galloyl-beta-D-glucopyranose | -0.0117 | -0.2928 | 0.1198 | 0.2599 | -0.2026 | 0.2803 | -0.1644 | 0.2214 | -0.462^*^ | 0.1198 | 0.2197 |
| 3, 5-Cyclo-5alpha, 17alpha-pregn-20-yne-6beta, 17-diol | 0.1458 | -0.2948 | 0.3653^*^ | 0.2896 | -0.0668 | 0.2744 | -0.2688 | 0.1202 | -0.0194 | 0.3653^*^ | 0.0484 |
| Goshuyic acid | 0.2287 | -0.343^*^ | 0.172 | 0.0721 | -0.0545 | 0.2765 | -0.098 | 0.2905 | 0.0335 | 0.172 | 0.1359 |
| Methyl 3- (2, 3-dihydroxy-3-methylbutyl) -4-hydroxybenzoate | 0.1868 | -0.401^*^ | 0.2023 | 0.2082 | -0.0683 | 0.1611 | -0.0838 | 0.3564^*^ | -0.1551 | 0.2023 | 0.0792 |
| 2- (3-methylbut-2-en-1-yl) benzene-1, 3, 5-triol | 0.2465 | -0.340^*^ | 0.2346 | 0.2234 | -0.0537 | 0.2467 | -0.1094 | 0.3658^*^ | -0.0878 | 0.2346 | 0.0814 |
| Isobutyrylglycine | 0.0452 | -0.2953 | 0.1699 | 0.0665 | -0.0338 | 0.4172^*^ | -0.1374 | 0.1425 | -0.339^*^ | 0.1699 | 0.1184 |
| Pyrraline | 0.0684 | -0.377^*^ | 0.1055 | 0.1986 | -0.1841 | 0.1194 | -0.1481 | 0.2351 | -0.3379 | 0.1055 | 0.0598 |
| 3, 4, 5-Trimethoxyphenyl acetate | 0.2057 | -0.424^*^ | 0.3166 | 0.2418 | -0.2014 | 0.1603 | 0.0025 | 0.3288 | -0.1714 | 0.3166 | 0.1839 |
| Garcinia lactone dibutyl ester | 0.1809 | -0.503^*^ | 0.2695 | 0.2483 | -0.1822 | 0.1618 | -0.0751 | 0.2631 | -0.1865 | 0.2695 | 0.2762 |
| 2- [4, 6-dihydroxy-2-methoxy-3- (3-methylbut-2-en-1-yl) phenyl] acetic acid | 0.1856 | -0.507^*^ | 0.292 | 0.2025 | -0.2376 | 0.1828 | -0.0305 | 0.2498 | -0.2438 | 0.292 | 0.272 |
| 2-Benzyl-4, 5-dimethyl-1, 3-dioxolane | 0.2717 | -0.434^*^ | 0.2299 | 0.1996 | -0.1361 | 0.2483 | -0.0692 | 0.2178 | -0.2155 | 0.2299 | 0.3725^*^ |
| 6- (1-Hydroxyethyl) -2, 2-dimethyl-2H-1-benzopyran | 0.2063 | -0.393^*^ | 0.3201 | 0.3485^*^ | -0.1294 | 0.2512 | 0.0471 | 0.3167 | -0.1564 | 0.3201 | 0.336 |
| Lysyl-Tyrosine | 0.2119 | -0.390^*^ | 0.3015 | 0.2291 | -0.2056 | 0.2962 | -0.1962 | 0.2763 | -0.123 | 0.3015 | 0.2297 |
| Cortolone-3-glucuronide | 0.3187 | -0.3201 | 0.4144^*^ | 0.2265 | -0.2981 | 0.2881 | -0.1555 | 0.2188 | -0.1485 | 0.4144^*^ | 0.2392 |
| Raffinose | 0.2113 | -0.3206 | 0.3565^*^ | 0.2503 | -0.303 | 0.3249 | -0.1228 | 0.1278 | -0.1974 | 0.3565^*^ | 0.1737 |
| { [3- (2, 5-dihydroxyphenyl) prop-2-en-1-yl] oxy} sulfonic acid | 0.2548 | -0.3087 | 0.3825^*^ | 0.3237 | -0.3133 | 0.2563 | -0.2731 | 0.3548^*^ | -0.2204 | 0.3825^*^ | 0.2633 |
| O-Adipoylcarnitine | 0.2501 | -0.399^*^ | 0.3957^*^ | 0.3564^*^ | -0.2968 | 0.2675 | -0.1381 | 0.3203 | -0.1802 | 0.3957^*^ | 0.3076 |
| Lepidimoic acid | 0.2521 | -0.348^*^ | 0.3291 | 0.3987^*^ | -0.3202 | 0.2458 | -0.1379 | 0.2474 | -0.2076 | 0.3291 | 0.2833 |
| Glutaminylproline | 0.1931 | -0.353^*^ | 0.3491^*^ | 0.3053 | -0.360^*^ | 0.1615 | -0.1535 | 0.3755^*^ | -0.3301 | 0.3491^*^ | 0.1144 |
| Asteltoxin | 0.2636 | -0.419^*^ | 0.3702^*^ | 0.2843 | -0.3195 | 0.2706 | -0.0551 | 0.2983 | -0.350^*^ | 0.3702^*^ | 0.3008 |
| Etiocholanolone | -0.004 | -0.418^*^ | 0.2042 | 0.2359 | -0.2408 | 0.0564 | -0.1081 | -0.0113 | -0.1871 | 0.2042 | 0.2281 |
| 4-Androstenediol | 0.008 | -0.348^*^ | 0.2635 | 0.2109 | -0.2663 | 0.0589 | -0.062 | 0.0126 | -0.1554 | 0.2635 | 0.2945 |
| Traumatic Acid | 0.189 | -0.461^*^ | 0.201 | 0.2138 | -0.0799 | 0.2061 | -0.0764 | 0.1159 | -0.1722 | 0.201 | 0.1523 |
| PS (16:1 (9Z) /22:5 (7Z, 10Z, 13Z, 16Z, 19Z) ) | 0.2245 | -0.2284 | 0.3484^*^ | 0.1994 | -0.2482 | 0.234 | -0.1024 | 0.0818 | -0.0186 | 0.3484^*^ | 0.2102 |
| 1, 11-Undecanedicarboxylic acid | 0.2025 | -0.1808 | 0.2672 | -0.0685 | -0.1509 | 0.3505^*^ | 0.2329 | 0.1865 | 0.0076 | 0.2672 | 0.2096 |
| Yuzu lactone | 0.1301 | -0.1232 | 0.2797 | -0.1102 | -0.0641 | 0.2707 | 0.1714 | 0.1131 | 0.0304 | 0.2797 | 0.3437^*^ |
| 3alpha-Hydroxyoreadone | 0.1788 | -0.353^*^ | 0.2397 | 0.1278 | -0.0774 | 0.3214 | 0.0072 | 0.1907 | 0.0281 | 0.2397 | 0.4659^*^ |
| 3, 11, 12-Trihydroxy-1 (10) -spirovetiven-2-one | 0.37^*^ | -0.2611 | 0.1838 | 0.1996 | -0.1575 | 0.3455^*^ | 0.1052 | 0.1647 | -0.0304 | 0.1838 | 0.3694^*^ |
| PS (20:2 (11Z, 14Z) /20:0) | 0.37^*^ | 0.0107 | 0.3059 | 0.1582 | -0.1438 | 0.3137 | 0.129 | 0.3379 | 0.0073 | 0.3059 | -0.048 |
| Cholic acid glucuronide | 0.2703 | -0.2135 | 0.1201 | 0.1462 | -0.0148 | 0.5544^*^ | -0.0828 | 0.2661 | -0.0061 | 0.1201 | 0.0645 |

**P* value <0.05
